# Hyperglycemia in Diabetes Drives Hepatic Fibrosis Through the Non-Enzymatic Function of HK2 and AGEs-mediated Matrix Stiffening

**DOI:** 10.7150/ijbs.130351

**Published:** 2026-08-24

**Authors:** Chuxin Huang, Jiali Qian, Li Zhang, Ying Liu, Qiwen Zhou, Jiajun Li, Yingying Zhao, Hongmei Yu, Yawen Zhang, Jinya Huang, Miao Li, Ziyu Ren, Zihao Xu, Rui Liu, Xuanchun Wang, Jie Chen, Jiefeng Cui, Yehong Yang

**Affiliations:** 1 Department of Endocrinology, Huashan Hospital, Fudan University, Shanghai 200040, China.; 2 Liver Cancer Institute, Zhongshan Hospital, Fudan University & Key Laboratory of Carcinogenesis and Cancer Invasion, Ministry of Education, Shanghai 200032, China.; 3 Department of General Surgery, Comprehensive Breast Health Center, Ruijin Hospital, Shanghai Jiao Tong University School of Medicine, Shanghai, P. R. China.; 4 Department of Gastroenterology and Hepatology, Zhongshan Hospital, Fudan University, Shanghai, China.

**Keywords:** empagliflozin, diabetes mellitus, hepatic fibrosis, hexokinase 2, signal transducer and activator of transcription 3, advanced glycation end products

## Abstract

Diabetes is implicated in accelerating the progression of hepatic fibrosis, and empagliflozin (an SGLT2 inhibitor) has potential therapeutic effects on diabetic hepatic fibrosis. However, their underlying mechanisms are still largely uncharacterized. In this study, we found that diabetes obviously promoted hepatic fibrosis progression, and empagliflozin intervention effectively impeded this pathological process independent of its hypoglycemic effects. Mechanistically, high glucose stimulation significantly increased HSC activation and its metabolic enzyme HK2 dissociation from mitochondria. The dissociated HK2 interacted with STAT3 to increase its nuclear entry for upregulating HSC activation-associated gene expression. Meanwhile, high glucose stimulation also suppressed KLF4 expression to attenuate its inhibitory effect on STAT3 transcription activation in HSCs. Additionally, high glucose-induced AGEs accumulation resulted in matrix stiffening, which also increased HSC activation. Empagliflozin alleviated hepatic fibrosis progression through decreasing AGEs accumulation and suppressing STAT3 and FOXO1 phosphorylation in HSCs. From the new perspectives of the non-metabolic functions of metabolic enzymes and matrix biomechanics, this study sheds light on a new synergistic regulatory pathway through which diabetes promotes hepatic fibrosis and highlights empagliflozin as a potential therapeutic agent for diabetic hepatic fibrosis.

## Introduction

Both type 1 and type 2 diabetes are significantly associated with an increased risk of hepatic fibrosis 1,2. The prevalence rates of advanced hepatic fibrosis and cirrhosis in adult patients with type 2 diabetes (T2DM) range from 14% to 15.4% and 6% to 7.7%, respectively 3,4. A prospective study involving 500,000 participants reported that individuals with diabetes have a 1.81-fold increased risk of developing cirrhosis compared to those without diabetes 1. Diabetes is also an independent prognostic factor for unfavorable outcomes in patients with cirrhosis 2. These data suggest a close association between diabetes and hepatic fibrosis, with hyperglycemia potentially contributing to the progression of hepatic fibrosis.

Diabetes-induced hepatic fibrosis is generally attributed to oxidative stress 5,6, inflammatory responses 7,8, insulin resistance 5,9, and advanced glycation end products (AGEs) 10,11. Besides, high glucose stimulation also directly activated hepatic stellate cells (HSCs) and thereby contributes to hepatic fibrosis occurrence 6,12. However, the underlying mechanisms by which high glucose activates HSCs, particularly through the non-enzymatic functions of metabolic enzymes and biomechanical regulation, remain largely unknown. Under normal physiological conditions, glycolytic enzymes such as hexokinase (HK), phosphofructokinase-1 (PFK-1), and pyruvate kinase (PK), are primarily involved in aerobic glycolysis 13. However, under some pathological conditions, glycolytic enzymes can perform non-metabolic functions, different from classical metabolic enzyme functions 14-16. Glycolytic enzyme PFK-1 exhibits a non-metabolic function to influence ZEB1 lactylation, and thereby suppress the proliferation and metastasis of bladder cancer cells 17. Under high glucose stimulation, PKM2 undergoes nuclear translocation to downregulate chemokine expression through interacting with TRX1, and subsequently reinforces an immunosuppressive microenvironment to promote hepatocellular carcinoma (HCC) metastasis 16. Besides, in human glioblastoma cells, high glucose stimulation dissociates HK2 from mitochondria to the cytoplasm, phosphorylates IκBα to increase PD-L1 expression and immune evasion 18. Accordingly, non-metabolic functions of metabolic enzymes indeed exert important effects on cellular functional status and various pathological events. On the other hand, AGEs, as the product of long-term hyperglycemia in diabetes, also alter the physical and biomechanical properties of the extracellular matrix (ECM) in the liver 19-21, potentially promoting the activation of HSCs. Hereby, we speculated that there may be a synergistic regulatory mechanism in high glucose-activated HSCs between non-metabolic function of metabolic enzyme and AGEs-caused matrix stiffening.

Empagliflozin, as a selective inhibitor of sodium-glucose cotransporter 2 (SGLT2), can effectively lower blood glucose levels by promoting urinary glucose excretion 22. Recent studies indicate that SGLT2 inhibitors also exert anti-fibrotic effects in the liver 23,24. In a multicenter, randomized controlled trial 25, SGLT2 inhibitors obviously ameliorated the degree of hepatic fibrosis in T2DM patients with metabolic dysfunction-associated fatty liver disease (MAFLD) through glucose-lowering and improvement of insulin sensitivity 23,24. Similarly, SGLT2 inhibitors significantly alleviate hepatic steatosis and hepatic fibrosis progression in patients with MAFLD through body weight loss 26,27. Notably, dapagliflozin has recently been reported to improve hepatic fibrosis in a bile duct ligation-induced hepatic fibrosis rat model without diabetes or hepatic steatosis, implying that SGLT2 inhibitors may directly prevent hepatic fibrosis through new mechanisms independent of glucose lowering and fat reduction 28. However, the precise mechanism underlying the direct effects of SGLT2 inhibitors on hepatic fibrosis remains unclear.

In this study, we uncovered a synergistic regulatory mechanism underlying diabetes-induced hepatic fibrosis progression and proposed that high glucose promoted HSC activation to accelerate hepatic fibrosis through the non-metabolic function of HK2 and AGEs-induced matrix stiffening. Additionally, therapeutic effects of empagliflozin on hepatic fibrosis were also validated via glucose-lowering and non-glucose-lowering pathways. Our findings provide new insights into the synergistic regulatory pathways through which diabetes promotes hepatic fibrosis and highlight empagliflozin as a potential agent of empagliflozin for diabetic hepatic fibrosis.

## Materials and methods

### Clinical human hepatic fibrosis specimens

Clinical data of 36 hepatic fibrosis patients who underwent liver biopsy in Zhongshan Hospital were collected. The inclusion criteria were as follows: (1) age 18-75 years; (2) diagnosis of MASLD according to the 2023 criteria with baseline liver fibrosis assessment (liver stiffness measurement by transient elastography or FIB-4 index); (3) availability of complete baseline data on fasting glucose, HbA1c, ALT, and AST; (4) provision of signed informed consent; (5) for diabetic patients, inclusion followed ADA criteria (fasting plasma glucose ≥ 7.0 mmol/L or HbA1c ≥ 6.5%). Exclusion criteria included: (1) other chronic liver diseases (hepatitis B, hepatitis C, autoimmune hepatitis, drug-induced liver injury); (2) excessive alcohol consumption (> 30 g/day for men, > 20 g/day for women) for at least one year; (3) use of hepatotoxic drugs; (4) severe cardiac or renal insufficiency, malignancy, or pregnancy; and (5) contraindications to elastography. The patients were divided into two groups according to their blood glucose and HbA1c levels: the hepatic fibrosis group (n = 18) and the hepatic fibrosis + DM group (n = 18). Clinical characteristics, blood glucose levels, AGEs, and liver stiffness were compared among the groups, and the correlations among these parameters were further analyzed. In addition, immunofluorescence co-staining was performed to examine the expression of pFOXO1 and pSTAT3 in HSCs within from liver tissues in each group. All participants provided written informed consent. The study was approved by the Zhongshan Hospital Research Ethics Committee (Approval No. B2025-635R).

### Animals and experimental design

5-weeks-old male C57BL/6J mice were obtained from Shanghai Jihui Laboratory Animal Breeding Co., Ltd and housed in the Department of Laboratory Animal Science, Fudan University (Shanghai, China) under standard conditions with a 12-hour light/dark cycle, at a constant temperature of 22 ± 2°C, with free access to water and food. After one-week acclimatization, mice were randomly assigned to two groups: normal chow diet (NCD) group (n = 5) and high-fat diet (HFD) group (n = 24). After 25 weeks, mice in the HFD group were intraperitoneally injected with STZ (40 mg/kg/day, dissolved in citrate buffer) for five consecutive days, while mice in NCD group were intraperitoneally injected with an equal volume of citrate buffer as a solvent control. Random blood glucose levels were subsequently measured weekly, and mice injected with STZ showing random blood glucose levels exceeding 16.7 mmol/L were included for further experimentation at week 32. Subsequently, the experimental groups were as follows: (1) The NCD group served as the Control group (n = 5); (2) T2DM group (n = 6); (3) T2DM+Insulin group (n = 9); (4) T2DM+Empagliflozin group (n = 9). Mice in T2DM+Insulin group were subcutaneously injected with Insulin (0.2-2U/kg/d), while Mice in T2DM+Empagliflozin group were orally administered empagliflozin (10mg/kg/d) for 6 weeks. Insulin dose (0.2-2 U/kg/d) was adjusted to maintain comparable blood glucose levels between the T2DM+Insulin group and the T2DM+ Empagliflozin group throughout the intervention period. After treatments, mice were sacrificed and liver samples were harvested for further analysis. All procedures were approved by the Animal Ethics Committee of Fudan University (Shanghai, China).

### Oral glucose tolerance test (OGTT) and intraperitoneal insulin tolerance test (IPITT)

For OGTT, mice were fasted for 12 hours and then orally administered 2 g/kg glucose. Blood samples were collected from the tail vein at 0, 15, 30, 60, 90 and 120 minutes post-glucose administration to measure blood glucose levels. For the IPITT, mice were fasted for 4 hours and subsequently received an intraperitoneal injection of 1 U/kg insulin. Blood glucose levels were measured at 0, 15, 30, 60, 90 and 120 minutes after insulin injection. The area under the curve (AUC) for both OGTT and IPITT was calculated based on the blood glucose data obtained.

### Histological staining

After the experimental treatments, liver tissues were collected and processed for histological analysis. The tissues were fixed in 4% paraformaldehyde and subsequently embedded in paraffin. Sections (5 μm thick) were prepared for histological examination. Hematoxylin and eosin (HE) staining was performed to assess the overall liver architecture and histopathological features. For the evaluation of collagen deposition and hepatic fibrosis, Masson's trichrome staining was carried out, which stains collagen fibers blue while the surrounding hepatic parenchyma appears red. Sirius red staining was also used to specifically identify collagen deposition, with positive areas displaying bright red birefringence under polarized light. Additionally, the positive areas of Masson's and Sirius red staining were quantified using ImageJ software.

### Immunohistochemistry (IHC) and immunofluorescence (IF) co-staining analysis

For IHC, formalin-fixed, paraffin-embedded tissue sections were deparaffinized, rehydrated, and subjected to antigen retrieval using an appropriate citrate buffer. After blocking with 10% goat serum (Solarbio, SL038), the sections were incubated with primary antibodies (Collagen type I, 1:500, Proteintech, 14695-1-AP; α-SMA, 1:1000, Signalway Antibody, 40482; TIMP1, 1:400, Proteintech, 16644-1-AP; AGEs, 1:200, Abcam, ab23722) overnight at 4 °C. After washing with PBS, the sections were treated with corresponding horseradish peroxidase-conjugated secondary antibodies and protein localization was visualized by applying the DAB detection method (Gene Tech, GK500710), followed by counterstaining with hematoxylin. IHC images were analyzed using ImageJ software to quantify the staining intensity.

For IF co-staining, liver tissue sections were first subjected to antigen retrieval as described above. After blocking non-specific binding with 10% goat serum, the sections were incubated overnight at 4 °C with a combination of primary antibodies (pFOXO1, 1:100, Cell Signaling Technology, 9461; α-SMA, 1:200, Signalway Antibody, 40482; pSTAT3, 1:100, Cell Signaling Technology, 9145). The sections were then incubated with fluorophore-conjugated secondary antibodies (Alexa Fluor 594 goat anti-mouse, 1:200, YEASEN, 34112ES60; Alexa Fluor 488 goat anti-rabbit, 1:200, YEASEN, 34206ES60) for 1 hour at room temperature. To visualize cell nuclei, sections were counterstained with DAPI (Beyotime, C1002). Fluorescent signals were captured using a fluorescence microscope (Nikon Eclipse C1), and co-localization of the target proteins was assessed.

### Reverse transcription quantitative polymerase chain reaction (RT-qPCR)

Total RNA was extracted from liver samples or HSCs using TRIzol reagent (Invitrogen). The quality and concentration of the RNA were assessed using a NanoDrop 2000 spectrophotometer (Thermo Fisher Scientific, USA). cDNA synthesis was carried out according to the manufacturer's instructions, followed by qPCR using the SYBR Green PCR system (YEASEN, 11204ES08). Gene expression levels were analyzed using the 2^-ΔΔCt^ method. All primers used in the study were synthesized by Sangon Biotech (Shanghai, China). The sequences of the primers used in this study are listed in Supplementary Table S1-S2.

### Western blot

Total proteins were extracted from tissue or cell samples using RIPA lysis buffer (Beyotime, P0013B), containing protease (Beyotime, P1005) and phosphatase inhibitors (Beyotime, P1081). Nuclear proteins were isolated using a nuclear and cytoplasmic extraction kit (Thermo Fisher Scientific, 78833) according to the manufacturer's protocol. Mitochondrial proteins were extracted using a mitochondrial isolation kit (Beyotime, C3601). The protein concentration was quantified using the BCA Protein Assay Kit (Beyotime, P0010) according to the manufacturer's instructions. Subsequently, protein samples were separated by SDS-PAGE and transferred to a PVDF membrane. The membrane was blocked with QuickBlock™ Blocking Buffer (Beyotime, P0220) and then incubated with the primary antibody at 4 °C overnight. Afterward, the membrane was incubated with HRP-conjugated secondary antibody at room temperature for 1 hour. Detection was performed using a chemiluminescent substrate (YEASEN, 36208ES60), and quantitative analysis was conducted using ImageJ software. The primary antibodies were diluted according to the following concentrations: Collagen type I (1:1000, Proteintech, 67288-1-Ig), α-SMA (1:5000, Signalway Antibody, 40482), TIMP1 (1:1000, Proteintech, 16644-1-AP), β-actin (1:20000, Proteintech, 66009-1-Ig), HK2 (1:1000, Abcam, ab209847), VDAC1 (1:1000, Proteintech, 55259-1-AP), STAT3 (1:1000, Cell Signaling Technology, 9193), p-STAT3 (1:1000, Cell Signaling Technology, 9145), PI3K (1:5000, Proteintech, 60225-1-Ig), p-PI3K (1:1000, Abmart, TA3242), AKT (1:1000, Cell Signaling Technology, 9272), p-AKT (1:1000, Cell Signaling Technology, 9271), FOXO1 (1:1000, Proteintech, 18592-1-AP), pFOXO1 (1:1000, Cell Signaling Technology, 9461T), KLF4 (1:1000, Proteintech, 11880-1-AP), AGEs (1:1000, Abcam, ab238539), Integrin αV (1:1000, Cell Signaling Technology, 4711), Integrin β5 (1:1000, Cell Signaling Technology, 4708), SGLT2 (1:1000, Santa Cruz Biotechnology, sc-393350), Histone-H3 (1:1000, Cell Signaling Technology, 9715), Tubulin (1:1000, Proteintech, 66031-1-Ig), Flag (1:5000, PTM BIO, PTM-6075).

### Enzyme-linked immunosorbent assay (ELISA)

100 mg of mouse liver was homogenized in PBS and stored at -20°C overnight. After two cycles of freeze-thaw treatment to disrupt the cell membranes, the homogenate was centrifuged for 5 minutes (4 °C, 5000 g), and the supernatant was collected. The levels of AGEs in the liver tissue of mice from each group were quantified following the manufacturer's instructions for the AGEs assay kit (CUSABIO, China, CSB-E09414m). For type I mouse tail collagen gel samples, gel samples were transferred into microcentrifuge tubes, followed by homogenization and centrifugation to collect the supernatant after the intervention. Subsequently, the AGEs levels in the gel were measured using an AGEs ELISA kit (CLOUND-CLONE CORP, Wuhan, China, CEB353Ge), in accordance with the manufacturer's instructions. Optical density (OD) values were measured using a microplate reader at a wavelength of 450 nm.

### Cell culture and transfection

Human hepatic stellate cells (LX-2) and primary mouse HSCs were purchased from Hunan Fenghui Biotechnology (Hunan, China), while the hepatocyte (THLE-2) and human renal tubular epithelial cells (HK-2) were obtained from Procell (Wuhan, China). The cells were cultured in Dulbecco's Modified Eagle Medium (DMEM; Gibco, 11885084) supplemented with 10% fetal bovine serum (FBS; Gibco, A5670701) and 1% penicillin/streptomycin (Gibco, 15140122), and incubated at 37 °C in a 5% CO₂ atmosphere. In accordance with relevant studies on diabetes 16,29,30, glucose concentrations of 5.5 mM (normal glucose, NG), 11 mM (moderate glucose, MG), and 25 mM (high glucose, HG) were applied to represent normal, moderate, and high glucose conditions, respectively. Transfection was conducted using Lipofectamine 3000 Transfection Reagent (Invitrogen), following the manufacturer's protocol. The wild-type HK2, site-mutated HK2 (Flag-HK2-D793/K873A and Flag-HK2-E165/K191A), STAT3, and KLF4 overexpression plasmids were purchased from Shanghai Jikai Gene Medicine Co., Ltd. Detailed plasmid information is provided in Supplementary Table S3.

### Immunocytochemistry (ICC) and mitochondrial immunofluorescence co-localization

Cells were cultured in 24-well plates at an appropriate density. Following the intervention, the cells were fixed with 4% paraformaldehyde, permeabilized with 0.3% TritonX-100, and blocked with 10% goat serum. Subsequently, the cells were incubated at 4°C overnight with the primary antibodies (Collagen type I, 1:200, Proteintech, 67288-1-Ig; α-SMA, 1:200, Signalway Antibody, 40482; TIMP1, 1:200, Proteintech, 16644-1-AP; SGLT2, 1:50, Santa Cruz Biotechnology, sc-393350). The cells were subsequently incubated at room temperature for 1 hour with fluorophore-conjugated secondary antibodies (Alexa Fluor 594 goat anti-mouse, 1:200, YEASEN, 34112ES60; Alexa Fluor 594 goat anti-rabbit, 1:200, YEASEN, 33112ES60). Nuclei were counterstained with DAPI (Beyotime, C1002) and fluorescent signals were acquired using a Nikon Eclipse C1 fluorescence microscope.

For mitochondrial immunofluorescence co-localization, following treatment with different glucose concentrations (NG, MG, HG), the cells were incubated with 200 nM Mito-Tracker Red CMXRos working solution (Beyotime, C1049B) at 37 °C for 30 minutes to label mitochondria. Subsequently, fluorescence staining was performed according to the above ICC protocol (HK2, 1:200, Abcam, ab209847). Imaging was conducted using a confocal fluorescence microscope (Nikon Eclipse C1), and immunofluorescence co-localization analysis was performed using ImageJ. Co-localization curves were generated using GraphPad Prism.

### Co-immunoprecipitation (Co-IP) analysis

Lysis buffer (Beyotime, P0013) was used to extract HSC lysates for Co-IP analysis. Approximately 1% of the lysate was retained as an input control, while the remaining lysate (containing approximately 1 mg of protein) was incubated overnight at 4 °C with the corresponding capture antibodies (HK2, Abcam, ab209847; STAT3, Cell Signaling Technology, 9193; KLF4, Proteintech, 11880-1-AP; Flag, PTM BIO, PTM-6075) under continuous rotation. On the second day, the antibody-protein complexes were incubated with 40 μL of protein A/G magnetic beads (Millipore, LSKMAGAGAG02) at room temperature for 1 hour with continuous rotation. The beads were then captured using a magnet, and the supernatant was carefully discarded. The beads were washed three times, and the proteins were eluted by boiling in 1× loading buffer for 10 minutes. Subsequently, the resulting eluates were analyzed by Western blotting.

### Co-IP coupled with LC-MS/MS analysis

The cytoplasmic proteins excluding mitochondria from HSCs cultured in high glucose (HG) conditions were extracted using a mitochondrial and cytoplasmic protein isolation kit (Beyotime, C3601). IP was performed using either the HK2 capture antibody or a negative control IgG antibody, followed by protein separation via SDS-PAGE. The protein bands were visualized using Coomassie blue staining, and the gel was sent to Shanghai Zhongke Biotech Company for LC-MS/MS analysis. Briefly, the gel was reduced and alkylated, and the proteins were digested with trypsin. The resulting peptides were analyzed using nanoLC-QE, and the mass-to-charge ratios of the peptides and peptide fragments were recorded. The raw data from mass spectrometry were processed using Proteome Discoverer 1.4 software and searched against the UniProt Homo sapiens database to obtain the final protein identification results.

### Nuclear proteomics analysis

Nuclear proteins from hepatic stellate cells after NG or HG treatment were extracted using a nuclear and cytoplasmic extraction kit, and the samples were sent to Shanghai Zhongke Biotech Company for mass spectrometry analysis. Briefly, the extracted proteins were enzymatically digested into peptides, which were then separated by liquid chromatography. The separated peptides were analyzed by MS. The collected MS data were processed using Mascot software for protein identification and quantitative analysis.

### HumanTFDB transcription factor prediction Analysis

Potential transcription factors for genes (*Col1a1*, *Timp1*, *Fn*, *Mmp2*, and *Vim*) were predicted using the HumanTFDB online database (http://bioinfo.life.hust.edu.cn/HumanTFDB#!/). The intersection of the predicted transcription factors was analyzed to identify common transcription factors regulating the activation of HSCs related genes.

### Molecular docking analysis

The crystal structures of HK2 and STAT3 proteins were retrieved from the PDB database. The ligands and receptors were prepared using the LigPrep module and Protein Preparation Wizard in Schrödinger. Molecular docking was then performed using Schrödinger's Glide module. Glide predicts the binding mode between the ligand and receptor by generating various conformations and rotational states of the ligand at the receptor binding site. The most probable binding mode was selected based on docking scores and conformations, and different binding modes were ranked and quantitatively analyzed.

### DNA pull down

The promoter regions of the genes *Col1a1* (NM_000088.4), *Timp1* (NM_003254.3) and *Klf4* (NM_004235.6) were retrieved from the NCBI database (https://www.ncbi.nlm.nih.gov/). The binding sequences of STAT3 to the promoters of *Col1a1* and *Timp1*, as well as FOXO1 in the promoter region of Klf4, were predicted using the online tool Jaspar (https://jaspar.elixir.no/), with the prediction results shown in Supplementary Table S4-6. Based on these predictions, biotin-labeled DNA probes containing the highest score and relative score sequences, as well as biotin-free negative probes, were designed and synthesized by GenScript. The specific probe sequences are listed in Supplementary Table S7. DNA-protein interactions were then validated using a DNA pull-down kit (FITGENE, FI8901-24T). Briefly, 3 μg of DNA probes from the experimental and control groups were incubated with 40 μL of streptavidin magnetic beads at room temperature for 30 minutes. The mixture was then placed on a magnetic rack, and the supernatant was discarded. The corresponding group sample lysis buffer was added, and the mixture was incubated on a mixer at 4 °C for 2-4 hours. After incubation, the mixture was subsequently placed on a magnetic rack, and the supernatant was discarded. Subsequently 1×SDS-PAGE loading buffer was added, and the mixture was heated at 95 °C for 5 minutes. Afterward, it was placed on the magnetic rack and the supernatant was collected into a new centrifuge tube. The eluted products were used for western blot analysis.

### *In vitro* kinase assay

The *in vitro* kinase assay was performed as previously described 18. Briefly, bacterially purified recombinant HK2 (200 ng) and recombinant STAT3 protein (400 ng) were incubated in 40 μL kinase buffer containing 25 mM Tris-HCl (pH 7.5), 5 mM β-glycerophosphate, 2 mM dithiothreitol (DTT), 0.1 mM Na₃VO₄, and 10 mM MgCl₂, supplemented with 50 μM ATP-γ-S, at 30 °C for 30 min. The reaction was terminated by addition of 50 mM p-nitrobenzyl mesylate (PNBM) in 5% DMSO and incubated for 1 h at room temperature. Samples were then subjected to SDS-PAGE followed by immunoblotting. Phosphorylated STAT3 was detected using an anti-thiophosphate ester antibody.

### National health and nutrition examination survey (NHANES) database

Publicly available NHANES datasets of the population who underwent liver elastography or fasting blood glucose testing from 2017-2018 were downloaded. After merging datasets, a dataset of the population who underwent both liver elastography and fasting blood glucose testing was obtained. Based on the fasting blood glucose results, the population was divided into two groups: the normal group (FBG < 7 mmol/L) and the diabetes group (FBG ≥ 7 mmol/L). The difference in liver stiffness between the normal and diabetes groups was analyzed and compared.

### Glycation and gel preparation of type I collagen from mouse tail *in vitro*

Based on glucose concentration and the inhibition of AGEs formation, the experiment was divided into four groups: 0 mM, 50 mM, 100 mM, and 100 mM+ALT711. D-glucose was weighed according to the required concentration for each group, dissolved in type I collagen from mouse tail, and incubated at 4 °C for 10 days to induce glycation. Subsequently, type I collagen from mouse tail was used to prepare the gel according to the manufacturer's instructions (Corning, 354236). After the gel formed, the 100 mM + ALT711 group was treated with ALT711 for 1 hour.

### Measurement of stiffness and fiber arrangement of type I collagen mouse tail gel

The prepared type I collagen gel from mouse tail was used for subsequent stiffness determination using a texture profile analyzer. A force-time curve was plotted based on the variation of force over time during the measurement, and the gel fracture strength of each group was recorded. After *in vitro* glycation and preparation of type I collagen gel from mouse tail, the gel was fixed with 2.5% glutaraldehyde at 4 °C for 24 hours. Following standard dehydration and sputter coating procedures, the fiber arrangement of the type I collagen gel was visualized using a scanning electron microscope (JEOL, Tokyo, Japan) at an accelerating voltage of 2.0 kV in Fudan University.

### *In vitro* construction of FN-coated polyacrylamide gels with varying stiffness

As described in our previous study 31, we established FN-coated polyacrylamide gels with stiffness levels of 6 kPa (low stiffness) and 16 kPa (high stiffness) for cell culture. Cells were cultured on substrates with different stiffness and collected following the previously reported protocol 32,33.

### Intervention of LY294002, empagliflozin, ALT711, SB273005 *in vitro*

LY294002 (PI3K inhibitor, 20 μM, MCE, USA) and Empagliflozin (SGLT2 inhibitor, 79 μM, MCE, USA) were used to reverse high glucose-induced activation of HSCs to explore the role of the PI3K/AKT/FOXO1 pathway and the effect of SGLT2 inhibitors in diabetic hepatic fibrosis. The type I collagen from mouse tail was treated with ALT711 (AGE-breaker, MCE, USA) at a final concentration of 1 mM to investigate AGEs levels and collagen fiber arrangement after glycation of type I collagen. HSCs cultured on a 16 kPa stiffness matrix were treated with SB273005 (integrin αV/β5 inhibitor, Selleck, China) at a final concentration of 0.1 μmol/L 32.

### Construction of three-dimensional tissue-like model

HSCs were suspended in Matrigel on ice (4 °C) to ensure uniform cell distribution and then allowed to polymerize at 37 °C to establish a three-dimensional tissue-like culture system. After gelation, the constructs were maintained under standard culture conditions and subsequently exposed to NG, HG, or HG combined with empagliflozin treatment. Following the intervention period, the 3D cultures were carefully collected and processed for subcellular fractionation. Mitochondrial and cytoplasmic fractions were isolated according to standard protocols and to evaluate the subcellular localization of HK2.

### Statistical analysis

Experimental data are presented as mean ± standard deviation (SD). Statistical analyses and data visualization were performed using SPSS version 20 and GraphPad Prism 9.4.1 (GraphPad Software Inc., San Diego, CA, USA). Normally distributed data, as assessed by the Shapiro-Wilk normality test, were analyzed using Student's t-test for two-group comparisons or one-way analysis of variance (ANOVA) followed by Tukey's post hoc test for multiple-group comparisons, as appropriate. Non-normally distributed data were analyzed using the Mann-Whitney U test for two-group comparisons or the Kruskal-Wallis test followed by Dunn's multiple-comparisons test for multiple-group comparisons. A P-value of < 0.05 was considered statistically significant.

## Results

### Empagliflozin ameliorates hepatic fibrosis progression in high-fat diet/ streptozotocin (HFD/STZ)-induced T2DM mice

Referring to the methods described previously 34,35, we developed a T2DM mouse model with hepatic fibrosis to evaluate the role of hyperglycemia in hepatic fibrosis progression and the intervention effect of empagliflozin on this pathological progression. The experimental flow chart of animal model construction and empagliflozin intervention was shown in Fig. 1a. At the 32nd week, mice with blood glucose levels ≥ 16.7 mmol/L were randomly divided into three groups including the T2DM group, the T2DM + Insulin group, and the T2DM+Empagliflozin group (Fig. 1a and Fig. S1a-b). The mice in the T2DM+Insulin group were subcutaneously injected with insulin (0.2-2 U/kg/d, 6 weeks), while the mice in the T2DM+ Empagliflozin group were orally administered empagliflozin (10 mg/kg/d, 6 weeks) (Fig. 1a). Besides, the non-diabetic mice were defined as the control group. To eliminate the potential influence of hypoglycemia and weight loss on diabetic hepatic fibrosis, we set empagliflozin at a dose of 10 mg/kg/d and insulin at a dose of 0.2-2 U/kg/d for adjusting blood glucose levels to a similar level in both T2DM+Insulin and T2DM+Empagliflozin groups. During the 6-week intervention, we observed that blood glucose levels in both the T2DM+Insulin and T2DM+Empagliflozin groups remained at similar levels (Fig. S1c), and there were no significant changes in their body weight and food intake (Fig. S1d-f), suggesting that this experimental system can effectively minimize the influence of hypoglycemia and weight loss on diabetic hepatic fibrosis. Meanwhile, both insulin and empagliflozin significantly improved glucose tolerance and insulin resistance in T2DM mice (Fig. S1g-j). We comparatively analyzed the changes in liver tissue structure and fibrosis among the groups and observed that liver tissues in the T2DM group presented a disrupted hepatic lobule structure, indistinct boundaries, and marked fibrous tissue proliferation compared with liver tissues in the control group (Fig. 1b). On the contrary, liver tissues in both the insulin and empagliflozin intervention groups exhibited clearer hepatic lobule boundaries, more organized tissue structure, and reduced collagen fiber deposition, indicating hepatic fibrosis progression in HFD/STZ-induced T2DM mice is prominently prevented, especially in the T2DM+Empagliflozin group (Fig. 1b). Consistently, compared to the control group, liver tissues in the T2DM group exhibited significantly increased expression of Collagen type I, α-SMA, and TIMP1 at both the protein and gene levels. Simultaneously, compared to the T2DM group, liver tissues in two intervention groups all displayed obvious decreases in the expressions of Collagen type I, α-SMA, and TIMP1. Moreover, empagliflozin was superior to insulin in downregulating the expressions of these proteins (Fig. 1c-e). Collectively, diabetes significantly increases hepatic collagen deposition and promotes hepatic fibrosis progression. Empagliflozin effectively ameliorates hepatic fibrosis progression in HFD/STZ-induced T2DM mice. Notably, empagliflozin has obvious advantage over insulin in preventing the progression of diabetic hepatic fibrosis, indicating that in addition to its hypoglycemic effects, empagliflozin may have other mechanisms that impede the progression of diabetic hepatic fibrosis.

### High glucose stimulation increases HSC activation and its metabolic enzyme HK2 dissociation from mitochondria

The continuous activation of HSCs as the most important molecular event determines the progression of liver fibrosis 36. Once activated, HSCs will enhance collagen production and release a variety of cytokines, chemokines, and matrix metalloproteinases, which result in extracellular matrix proteins deposition and matrix remodeling to facilitate liver fibrosis progression 37. Considering that hyperglycemia and AGEs production are prominent manifestation of long-term diabetes 38,39, we speculate that high glucose environment and AGEs may directly or indirectly influence HSC activation. We first examined the effects of different concentrations of glucose on HSC activation, and observed that the expressions of HSC activation-associated proteins such as Collagen type I, α-SMA, and TIMP1 were progressively upregulated as glucose concentration was increased (Fig. 2a-c and Fig. S2b-d). Simultaneously, BAY-876 intervention (a glucose uptake inhibitor) significantly suppressed Collagen type I and TIMP1 expressions in HSCs and reversed this effect, confirming that high glucose stimulation has the ability to directly promote HSC activation (Fig. S2a). Glycolytic enzymes possess classical enzymatic functions and primarily participate in glucose aerobic oxidation under normal physiological conditions 40. However, under certain pathological conditions, glycolytic enzymes also have non-enzymatic functions and contribute to the development and progression of some diseases 14,16,41. Based on the roles of glycolytic enzyme HK2 in the activation of HSCs and the progression of hepatic fibrosis 42 and the finding that high glucose stimulation can induce the dissociation of HK2 from mitochondria to the cytoplasm in glioblastoma cells 18, we hypothesized that high glucose may stimulate HSC activation through altering the intracellular localization of HK2. We applied mitochondrial and cytoplasmic protein fractionation assay to examine the distribution of HK2 in the HSCs treated with different concentrations of glucose. The results displayed that high glucose stimulation prominently decreased the mitochondrial localization of HK2 but increased its cytoplasmic distribution. Moreover, high glucose did not alter the total protein level of HK2 (Fig. 2d). Besides, immunofluorescence staining analysis also revealed a marked reduction in mitochondrial localization of HK2 with the increase of glucose concentration (Fig. 2e). These data support that high glucose stimulation causes an obvious dissociation of HK2 from mitochondria to cytoplasm in HSCs, implying that HK2 dissociation may exhibit a non-enzymatic function and participate in high glucose-promoted HSC activation.

### Analysis of the binding protein with dissociated HK2 in high glucose-promoted HSC activation and differentially expressed nuclear proteins

To further explore the binding target protein with dissociated HK2 from the mitochondria in HSCs under high glucose stimulation, we applied immunoprecipitation (IP) combined with LC-MS/MS to identify the binding proteins with HK2 (Fig. 3a and Fig. S2e). We identified a total of 195 candidate HK2-binding proteins in the cytoplasm of HSCs under high glucose stimulation (Supplementary Table S8). Subsequently, we used the Human Transcription Factor Database (HumanTFDB) to predict the transcription factors for HSC activation-associated genes (including *Col1a1*, *Timp1*, *Fn*, *Mmp2* and *Vim*), and obtained 52 common transcription factors regulating the above genes (Fig. 3c and Supplementary Table S9). Considering the potential role of HK2-binding proteins in high glucose-activated HSC activation, we performed an intersection analysis between candidate HK2-binding proteins and predicted common transcription factors, and found the only common intersecting protein, signal transducer and activator of transcription 3 (STAT3) (Fig. 3b and Fig. 3d). We used Co-IP assay to further confirm the mutual binding between HK2 and STAT3 in the cytoplasm of HSCs (Fig. 3e-f). Since the contribution of STAT3 signaling pathway to HSC activation has been highlighted 43,44, the dissociated HK2 interacting with STAT3 may increase nuclear translocation of STAT3. Thereby, we further speculated that HK2 dissociation from mitochondria might interact with STAT3 and increase its nuclear translocation to improve HSC activation-associated gene expression. Additionally, it should be mentioned that other identified HK2-interacting proteins represent a broader exploratory interactome of HK2 under high glucose conditions, which may reflect additional metabolic and signaling adaptations.

On the other hand, we performed a comparative proteomic analysis of nuclear proteins extracted from HSCs under normal and high glucose stimulation for screening the target nuclear proteins related to HSC activation. We discovered 93 differentially expressed nuclear proteins, including 71 upregulated and 22 downregulated proteins in the high glucose stimulation group. Among the downregulated proteins, we determined KLF4 as a protein of interest for downstream analysis based on the following reasons: (1) Low expression of KLF4 is associated with HSC activation 45. (2) KLF4 was a significantly downregulated differential nuclear protein in high glucose stimulation group (Fig. 3g-h and Supplementary Table S10). (3) Nuclear KLF4 interacting with STAT3 can inhibit STAT3 transcriptional activity in adult retinal ganglion cells 46. Thus, high glucose stimulation may enhance STAT3-mediated HSC activation-associated gene transcription through downregulating KLF4 expression to weaken its inhibitory effect on the binding between STAT3 and promoter. The results in Fig. 3i-j further validated that high glucose stimulation downregulated the expression of nuclear and total KLF4. Meanwhile, we used Co-IP assays to examine the binding between STAT3 and KLF4 in the nuclear proteins of HSCs and observed that there existed a mutual binding between STAT3 and KLF4 (Fig. 3k-l). Given that HK2, as a kinase, is capable of phosphorylating its binding protein IκBα 18, and that STAT3 phosphorylation facilitates its nuclear translocation, we inferred that dissociated HK2 may phosphorylate STAT3 to increase its nuclear translocation, and thereby upregulate HSC activation-associated gene expression. In addition, high glucose-downregulated KLF4 expression suggested that its inhibitory effect on STAT3 binding to target gene promoters may be attenuated, thereby enhancing the expression of HSC activation-associated genes.

### Interaction between HK2 and STAT3 facilitates STAT3 phosphorylation and nuclear translocation to promote *Col1a1* and *Timp1* expressions

We further clarified whether high glucose stimulation facilitated STAT3 phosphorylation and its nuclear translocation to increase HSC activation. The results in Fig. 4a-b and Fig. S2f showed that high glucose significantly enhanced the phosphorylation and nuclear translocation of STAT3 in HSCs. Moreover, stattic (a STAT3 inhibitor) attenuated the expression of HSC activation-associated proteins induced by high glucose (Fig. S2i). We subsequently overexpressed HK2 to clarify the relationship between STAT3 phosphorylation and HK2 in HSCs under high glucose stimulation. We discovered that HK2 overexpression not only significantly increased the expression of Collagen type I and TIMP1, but also elevated STAT3 phosphorylation in HSCs (Fig. 4c). Moreover, HK2 overexpression also remarkably increased nuclear translocation of STAT3 in HSCs (Fig. 4d). Co-IP assay was used to investigate the phosphorylation level of STAT3 bound to HK2. The results demonstrated that HK2 overexpression obviously increased the phosphorylation levels of STAT3 bound to HK2, indicating that the interaction between HK2 and STAT3 can enhance STAT3 phosphorylation in HSCs (Fig. 4e). To confirm the promoting role of the interaction between HK2 and STAT3 in STAT3 phosphorylation and HSC activation, we performed a molecular docking analysis using Schrödinger software to predict the binding sites between HK2 and STAT3. The results showed that these two proteins had strong binding, and the potential binding sites were identified on HK2 at Lys873, Asp793, Lys191, and Glu165 (Fig. 4f-g). We constructed two HK2 mutant plasmids (Flag-HK2-D793/K873A and Flag-HK2-E165/K191A) and transfected them into HSCs. We examined the changes in HSC activation and STAT3 phosphorylation, and found that both HSCs transfected with Flag-HK2-WT and those with Flag-HK2-D793/K873A exhibited obvious increases in the expressions of Collagen type I and TIMP1, as well as STAT3 phosphorylation and its nuclear translocation, which was consistent with the above results. However, HK2 mutation (E165/K191) obviously reversed these effects (Fig. 4h-i). Furthermore, IP-western blot analysis supported that HK2 mutation (E165/K191) significantly reduced STAT3 phosphorylation level, and its nuclear translocation in HSCs, suggesting that the E165/K191 sites, rather than the D793/K873 sites, are the critical binding sites between HK2 and STAT3 (Fig. 4j). To confirm the direct role of HK2 in STAT3 phosphorylation, we performed an *in vitro* kinase assay and found that HK2 directly phosphorylated STAT3 (Fig. 4k), supporting a direct regulatory role of HK2 in STAT3 phosphorylation. Using the JASPAR database, we predicted the potential binding sequences of STAT3 in the promoter regions of *Col1a1* (Supplementary Table S4) and *Timp1* (Supplementary Table S5) genes. Based on the above binding sequences, we designed biotin-labeled DNA probes containing the highest scoring sequences, along with unlabeled negative control probes (Supplementary Table S6) for DNA pull-down assay. The result validated that STAT3 bound to the promoter regions of *Col1a1* and *Timp1* genes (Fig. 4l-m). Taken together, interaction between dissociated HK2 and STAT3 under high glucose stimulation effectively enhanced STAT3 phosphorylation and its nuclear translocation, and thereby increasing the expressions of *Col1a1* and *Timp1* in HSCs.

### High glucose stimulation downregulates KLF4 expression in HSCs and reduces its inhibitory effect on the binding of STAT3 to the promoter

As shown in Fig. 3j, high glucose stimulation significantly downregulated KLF4 expression in HSCs. Based on the reports that FOXO1 regulates KLF4 expression in B cells 47,48, and that high glucose stimulation activates PI3K/AKT/FOXO1 in renal tubular epithelial cells and pancreatic cells 49-51, we suppose that high glucose stimulation may activate the PI3K/AKT/FOXO1 pathway and inhibit nuclear translocation of FOXO1, thereby decreasing KLF4 expression in HSCs. We examined the effects of high glucose stimulation on the PI3K/AKT/FOXO1 pathway and observed that high glucose stimulation significantly enhanced the phosphorylation levels of PI3K, AKT and FOXO1 (Fig. 5a). In contrast, LY294002 (a PI3K inhibitor) intervention obviously reversed the phosphorylation levels of PI3K, AKT, FOXO1, simultaneously upregulated KLF4 expression and suppressed the expression of Collagen type I and TIMP1 in HSCs (Fig. 5b). Additionally, high glucose stimulation obviously attenuated FOXO1 nuclear translocation, and LY294002 intervention effectively reversed this effect (Fig. 5c-d). DNA pull-down assays revealed that FOXO1 specifically bound to the KLF4 promoter in HSCs, supporting a direct transcriptional regulation of KLF4 by FOXO1 (Fig. S2g). All these results strongly suggest that high glucose stimulation inhibits the nuclear translocation of FOXO1 by enhancing its phosphorylation level, and subsequently downregulates KLF4 to increase HSC activation. Previous study 46 and our data (Fig. 3k-l) all showed that there was an interaction between KLF4 and STAT3 within the nucleus of HSCs. We continued to explore the precise role of this interaction in HSC activation. We constructed two overexpression plasmids (Flag-STAT3-OE and Flag-KLF4-OE) to analyze their contribution to HSC activation. STAT3 overexpression significantly promoted the expressions of Collagen type I and TIMP1, but KLF4 overexpression had the opposite effect (Fig. 5e-f). We further performed DNA pull-down assay to assess the ability of STAT3 binding to the promoters of *Col1a1* and *Timp1* genes. The results showed that KLF4 overexpression reduced the binding of STAT3 to the promoters of both *Col1a1* and *Timp1*, suggesting that KLF4 inhibits STAT3's DNA-binding activity in HSCs (Fig. 5g-h). Overall, high glucose stimulation significantly inhibits FOXO1 nuclear translocation by increasing its phosphorylation, and thereby downregulates KLF4 expression. KLF4 downregulation diminishes its inhibitory effect on STAT3's DNA-binding activity, thereby enhancing STAT3-mediated HSC activation-associated genes expression.

### AGEs accumulation induced by high glucose stimulation increases matrix stiffening to promote HSC activation

In addition to the direct effect of high glucose, AGEs, the common products of long-term diabetes, also have a direct effect on HSC activation 52. Clinical studies have also demonstrated that AGEs accumulation is a significant risk factor for hepatic fibrosis 53. However, little is known about its regulatory mechanism in HSC activation. Recent studies have suggested a close association between increased matrix stiffness and HSC activation 54. Therefore, AGEs accumulation may influence matrix remodeling and mechanical properties to indirectly regulate HSC activation. We first examined the levels of AGEs in liver tissues of diabetic hepatic fibrosis mouse models, and found that the level of AGEs in T2DM group was obviously higher than that in the control group, revealing long-term diabetes can cause AGEs accumulation in liver tissue. Meanwhile, insulin and empagliflozin interventions prominently reduced hepatic AGEs levels (Fig. 6a), indicating that lowering blood glucose can decrease AGEs accumulation in liver tissue. Consistently, in ELISA and immunohistochemistry analysis, there was a significant increase in AGEs levels in the T2DM group, and a prominent decrease in AGEs accumulation in two intervention groups (Fig. 6b-c). These results indicate that AGEs accumulation may affect or participate in the progression of diabetic hepatic fibrosis. Using the NHANES database, we analyzed liver stiffness changes in patients with diabetes, and found that liver stiffness in diabetic individuals was remarkably higher than that in non-diabetic individuals, illustrating that diabetes may increase ECM stiffness in liver tissue (Fig. 6d).

We further validated whether the accumulation of AGEs increased ECM stiffness. We developed an experimental system *in vitro* (Fig. 6e) to evaluate glycation of type I collagen and its stiffness changes. The results showed that AGEs level in the collagen gel was obviously elevated with increase of glucose concentration, whereas ALT711 (an AGEs breaker) intervention decreased AGEs levels (Fig. 6f). Additionally, high glycation of collagen also increased the gel's stiffness, which was subsequently reversed by ALT711 intervention (Fig. 6g and Fig. S2h). These findings confirm that high glucose promotes the glycation of type I collagen, resulting an increase in AGEs levels and gel stiffness. To clarify how AGEs accumulation affects matrix stiffness, we employed scanning electron microscopy (SEM) to observe the fiber arrangement in the collagen gels treated with different glucose concentrations. We found that the collagen gel treated with high glucose exhibited a more curled and dense fiber arrangement, while ALT711 intervention partially restored a more regular fiber structure, suggesting that high glucose-induced AGEs accumulation increases matrix stiffness through making collagen fibers more tightly arranged (Fig. 6h).

Integrin αV/β5 has been validated to deliver mechanical stiffness signals into HSCs in our previous work 32. We speculated that increased matrix stiffness as an initiating contributor may influence STAT3 phosphorylation via Integrin αV/β5, subsequently leading to HSC activation. We established high- and low-stiffness substrates (16KPa and 6KPa) 55 to simulate fibrotic and normal liver tissue stiffness for assessing the effects of increased matrix stiffness on HSC activation. Compared with those in the low-stiffness group, the expression levels of Integrin αV/β5, Collagen type I, and TIMP1, as well as the phosphorylation level of STAT3 in the high-stiffness group were all distinctly elevated. Simultaneously, SB273005, an inhibitor of integrin αV/β5, significantly reversed the above changes of these molecules (Fig. 6i). All these data suggest that high glucose-induced AGEs accumulation can effectively enhance the level of matrix stiffness, which influences STAT3 phosphorylation to promote HSC activation.

### Empagliflozin alleviates diabetic hepatic fibrosis progression through decreasing AGEs accumulation and suppressing STAT3 and FOXO1 phosphorylation in HSCs

Based on the results in Fig. 1b-e, both insulin and empagliflozin interventions effectively prevented the progression of diabetic hepatic fibrosis. Moreover, empagliflozin was more effective than insulin in alleviating diabetic hepatic fibrosis, suggesting that empagliflozin may impede hepatic fibrosis progression through other pathways beyond its glucose-lowering and weight-reducing effects. Besides, high glucose-induced AGEs accumulation increased hepatic matrix stiffness, which also effectively promoted HSC activation. The data in Fig. 6a-i confirmed that empagliflozin also mitigated diabetic hepatic fibrosis through decreasing AGEs accumulation. To identify the specific cell in liver tissue targeted by empagliflozin, we used renal tubular epithelial cells as a positive control to examine the expression of SGLT2 in hepatocytes and HSCs. The results showed a significant expression of SGLT2 in LX-2 and primary murine HSCs, but a low expression in hepatocytes, indicating that empagliflozin exerts anti-fibrotic effect mainly by targeting HSCs (Fig. 7a-b and Fig. S2j). Based on a dose-response curve (Fig. 7c), we determined 79μM as the optimal intervention dose, which corresponds to approximately 80% cell viability in HSCs for evaluating its effect on HSC activation. We observed that empagliflozin significantly inhibited the expressions of Collagen type I and TIMP1 in HSCs and also decreased the phosphorylation levels of STAT3 and FOXO1 (Fig. 7d-e). Consistently, empagliflozin suppressed the nuclear translocation of STAT3, but promoted the nuclear translocation of FOXO1 in HSCs under high-glucose stimulation (Fig. 7f). Empagliflozin intervention also significantly reduced the dissociation of HK2 from mitochondria in HSCs (Fig. 7g). To further verify the dissociation of HK2 from mitochondria at the tissue level, we established a three-dimensional tissue-like HSCs model by embedding HSCs in Matrigel and treated them with high glucose and empagliflozin. Afterwards, we collected these models and extracted their mitochondrial and cytoplasmic fractions for analysis and found that high glucose stimulation indeed promoted the dissociation of HK2 from mitochondria to the cytoplasm. Moreover, empagliflozin intervention reversed this effect and restored HK2 mitochondrial localization (Fig. S2k), in agreement with the findings of cell experiment. These results strongly suggest that empagliflozin directly targets SGLT2 on HSCs, reverses the high glucose-induced HK2 dissociation from mitochondria, and inhibits the phosphorylation of STAT3 and FOXO1 to inactivate HSCs. Additionally, compared with those in the T2DM group, the phosphorylation levels of STAT3 and FOXO1 in HSCs were markedly reduced in the Empagliflozin group (Fig. 7h-i), supporting that empagliflozin alleviates hepatic fibrosis progression through suppressing STAT3 and FOXO1 phosphorylation. Collectively, empagliflozin alleviates diabetic hepatic fibrosis by lowering blood glucose levels and reducing AGEs accumulation. On the other hand, empagliflozin also directly suppresses HSC activation through suppressing the phosphorylation of STAT3 and FOXO1 to alleviate hepatic fibrosis.

### Clinical significance of diabetes in hepatic fibrosis progression

We collected and retrospectively analyzed the clinicopathological data of 36 hepatic fibrosis patients with or without diabetes (hepatic fibrosis group, n = 18, hepatic fibrosis+DM group, n = 18, Table 1) to further evaluate the clinical significance of diabetes in hepatic fibrosis progression. Analysis of clinical characteristics and pathological parameters showed that blood glucose levels of hepatic fibrosis patients with diabetes were significantly associated with the pathological degree of liver fibrosis (Fig. 8a-b). Additionally, liver stiffness of the hepatic fibrosis+DM group was remarkably higher than that of the hepatic fibrosis group (Fig. 8c). Correlation analysis also revealed a significant positive association between blood glucose level and either FIB-4 or liver stiffness (Fig. 8d-e), and HbA1c was also positively correlated with liver stiffness (Fig. 8f). We further measured serum AGEs levels in all 36 patients and analyzed their correlation with liver stiffness. The results demonstrated that serum AGEs level in the hepatic fibrosis + DM group were obviously higher than that in the hepatic fibrosis group, and AGEs level was also positively correlated with liver stiffness (Fig. 8g-h). Immunofluorescence staining was performed to assess the expressions of pFOXO1 and pSTAT3 in HSCs from the hepatic fibrosis group and hepatic fibrosis+DM group. Compared with the hepatic fibrosis group, the hepatic fibrosis + DM group exhibited significant increases in the levels of pFOXO1 and pSTAT3 in HSCs (Fig. 8i-j). These clinical data support that diabetes-associated hyperglycemia and AGEs contribute to the progression of diabetic hepatic fibrosis.

## Discussion

Although there exists a significant correlation between diabetes and hepatic fibrosis progression 2,56,57, the underlying mechanism by which high glucose stimulation as an initiating contributor regulates HSC activation remains largely unknown. SGLT2 inhibitors, a novel class of antidiabetic drugs, have shown significant effects in lowering blood glucose levels and body weight 58. Moreover, they may also exert great anti-fibrotic effects in diabetic hepatic fibrosis 25,59. However, little is known about the precise mechanism of the direct effects of SGLT2 inhibitors on hepatic fibrosis progression. Here, we successfully developed T2DM mouse models with hepatic fibrosis and evaluated the inhibitory effects of empagliflozin on the progression of hepatic fibrosis. Our results suggested that diabetes remarkably accelerated hepatic fibrosis progression. Moreover, empagliflozin intervention effectively impeded the progression of diabetic hepatic fibrosis independent of its glucose-lowering effects, implying that empagliflozin has additional anti-fibrotic intervention mechanism.

The continuous activation of HSCs determines the progression of hepatic fibrosis. Activated HSCs releases and secretes excessive ECM components, particularly type I collagen, which is considered the main cause of hepatic fibrosis progression 60,61. As an important risk factor, diabetes has been documented to promote hepatic fibrosis via multiple signaling pathways, including oxidative stress, AGEs, and the PI3K/AKT pathway 5,6,10,11. Similarly, high glucose stimulation also directly activates HSCs 12,62,63, and AGEs generated by long-term hyperglycemia can alter the physical and mechanical properties of the liver ECM 19,20,64, potentially promoting HSC activation. Recent research indicates that glycolytic enzymes have non-metabolic functions under specific pathological conditions, contributing to various intracellular biological activities, signal transduction, and cellular viability 14-16. Based on the above reasons, we speculate that there may be a synergistic regulatory mechanism between non-metabolic functions of metabolic enzymes and AGEs-induced matrix stiffening in diabetes-promoted hepatic fibrosis.

In this study, we first analyzed the effects of different concentrations of glucose on HSC activation, and observed that high glucose stimulation directly potentiated HSC activation, which was consistent with the findings* in vivo* that diabetes accelerates hepatic fibrosis. Simultaneously, high glucose stimulation also promoted the dissociation of metabolic enzyme HK2 from mitochondria to the cytoplasm in HSCs. Thus, the dissocitated HK2 dissociation from mitochondria is likely to participate in regulating HSC activation by its non-metabolic functions. Using an intersection analysis between the identified candidate HK2-binding proteins and the common transcription factors related to HSC activation-associated genes, we obtained the only common intersecting protein STAT3. Given the important role of STAT3 in HSC activation 43,44, we examined whether HK2 dissociation from mitochondria interacted with STAT3 to promote its nuclear translocation and upregulated HSC activation-associated gene expression. By STAT3 phosphorylation and nuclear translocation analyses, and binding site mutation experiments, we concluded that interaction between HK2 and STAT3 indeed increased STAT3 phosphorylation and its nuclear translocation, and the E165/K191 sites of HK2 were their important binding sites. DNA pull-down analysis further supported that STAT3 bound to the promoter regions of HSC activation-related genes *Col1a1* and *Timp1*. Based on the results of nuclear proteomics analysis and the potential inhibition role of KLF4 in the binding activity of STAT3 to the promoter of target gene 46, we selected KLF4 as a protein of interest to validated its role in high glucose-promoted HSC activation. Our results demonstrated that there was a mutual binding between STAT3 and KLF4 in the nucleus of HSCs and confirmed that high glucose stimulation promoted HSCs activation via suppressing KLF4 expression. Further experiments demonstrated that high glucose stimulation activated the PI3K/AKT/FOXO1 pathway to decrease nuclear translocation of FOXO1, and thereby suppressed KLF4 expression. Overall, high glucose stimulation can directly promote HSC activation through regulation of the HK2-STAT3 axis and its associated protein KLF4.

In addition to the direct regulatory mechanism, we also disclose an indirect regulatory mechanism in which AGEs generated by long-term diabetes enhance matrix stiffness, thereby stimulating HSC activation. AGEs are structural entities formed through non-enzymatic reactions between sugars and proteins, lipids, or nucleic acids 65. Prolonged hyperglycemia often accelerates the accumulation of AGEs, which in turn causes multifaceted damage to tissues and organs 66. Importantly, AGEs also induce irreversible alterations in the spatial structure of proteins, and their binding to collagen increases matrix rigidity and reduces matrix elasticity, thereby impairing the normal function of tissues 19,20. Therefore, AGEs formation may alter liver matrix stiffness to promote hepatic fibrosis progression. Our results showed that diabetic conditions significantly increased AGEs accumulation in the liver, and lowering blood glucose levels obviously reduced AGEs accumulation. Besides, glycation analysis of type I collagen revealed an obvious increase in AGEs formation, accompanied by elevated gel stiffness. Furthermore, glycated collagen fibers presented a more tightly curled and compact structure, which increased the stiffness level of the gel. A texture analyzer was used to evaluate the mechanical properties of collagen gels. This method indirectly reflects gel stiffness by measuring the resistance of the gel to deformation, and provides relatively uniform and reproducible measurements of bulk mechanical properties, allowing for consistent comparison across different experimental conditions. Subsequently, we cultured HSCs on high or low-stiffness substrates and observed that increased matrix stiffness upregulated STAT3 phosphorylation and contribute to HSC activation. Accordingly, a new synergistic regulatory mechanism was uncovered in high glucose-promoted HSC activation, and STAT3 is a common hub molecule in this regulatory process.

SGLT2 inhibitors mitigate the progression of diabetes-induced hepatic fibrosis, which is generally attributed to reduced renal tubular glucose reabsorption, lowering blood glucose levels, and improving insulin sensitivity 23,59,67. Thus, glucose-lowering therapy helps delay the progression of hepatic fibrosis 56. In our study, both insulin and empagliflozin treatment prominently alleviated hepatic fibrosis in diabetes, supporting that lowering blood glucose inhibits the progression of hepatic fibrosis. In diabetic patients, chronic hyperglycemia significantly accelerates the formation and accumulation of AGEs 68-70, which in turn promotes HSC activation 52. Our results also validated that prolonged hyperglycemia obviously increased AGEs accumulation and ECM stiffness in liver tissue, and therefore promoted HSC activation and hepatic fibrosis progression. Empagliflozin intervention reduced hepatic AGEs accumulation to mitigate their pro-fibrotic effects. Intriguingly, empagliflozin was more effective than insulin in alleviating diabetic hepatic fibrosis, indicating that empagliflozin also has additional anti-fibrotic intervention mechanism. Recent research demonstrates that dapagliflozin significantly reduces hepatic fibrosis in non-diabetic, non-fatty liver rats 28, also supporting that SGLT2 inhibitor may have non-hypoglycemic mechanisms to prevent diabetic hepatic fibrosis. Further experiments demonstrated that empagliflozin directly targeted HSCs to inhibit high glucose-induced HK2 translocation and its downstream STAT3 nuclear translocation, simultaneously promoting FOXO1 nuclear translocation, thereby jointly suppressing HSC activation. These findings sufficiently validate that empagliflozin can directly inhibit HSC activation, offering a novel intervention idea for hepatic fibrosis. Since hepatic fibrosis is a chronic and progressive disease, it should be acknowledged that the 6-week intervention period may not fully mirror the actual treatment cycle of empagliflozin treatment in clinical practice. However, our results and other reports 71,72 showed that 6-week intervention period was sufficient for observing therapeutic effects, indicating that there may be some differences in the treatment duration between animal models and clinical patients. Additionally, we observed a significant expression of SGLT2 in LX-2 cells and primary murine HSCs, but a low expression in hepatocytes, indicating that different glucose transporters may play dominant roles in different cell types. Hepatocytes may mainly rely on GLUT2-mediated glucose transport rather than SGLT2-mediated glucose uptake. However, activated HSCs during fibrogenesis exhibit metabolic characteristics distinct from hepatocytes. Activated HSCs undergo substantial metabolic reprogramming characterized by increased glucose utilization and may have alternative glucose transport pathways to support enhanced glycolytic activity. Previous studies have also reported SGLT2 expression in liver tissues and LX-2 cells 73-75. Therefore, SGLT2 expression in HSCs may reflect the specific metabolic demands of activated HSCs. But, the specific role of SGLT2 expression in HSCs still needs further investigation. Finally, we retrospectively analyzed clinical data to evaluate the clinical significance of diabetes in hepatic fibrosis progression and found that hyperglycemia and AGEs were closely associated with increased severity of hepatic fibrosis.

There may be some possible limitations in the study. A limitation is that only male mice were used in animal experiments, which may influence the generalizability of our findings to female subjects. The second limitation concerns the interpretation of clinical significance, as these results only reflect associations rather than causality. Future studies with larger cohorts and prospective designs are warranted to further confirm these causal relationships. Additionally, in the hepatic fibrosis + DM group, only 4 patients received insulin therapy and 3 patients received SGLT2 inhibitor therapy, with considerable differences in dosage and treatment duration among them. Due to the limited sample size and the variability in hypoglycemic drug dosage and treatment duration, we consider it is inappropriate to perform a meaningful analysis of its therapeutic effect on diabetes-related hepatic fibrosis.

In summary, this study uncovered a synergistic regulatory mechanism underlying diabetes-promoted hepatic fibrosis progression, and proposed that high glucose prominently promoted HSC activation and accelerated hepatic fibrosis through non-metabolic function of HK2 and AGEs-induced matrix stiffening, with STAT3 as a common hub molecule in this synergistic regulatory process. Additionally, empagliflozin plays an effective therapeutic role in diabetic hepatic fibrosis progression via hypoglycemic and non-hypoglycemic pathways, highlighting a potential therapeutic strategy for diabetic hepatic fibrosis.

## Supplementary Material

Supplementary figures and tables.

## Figures and Tables

**Figure 1:**
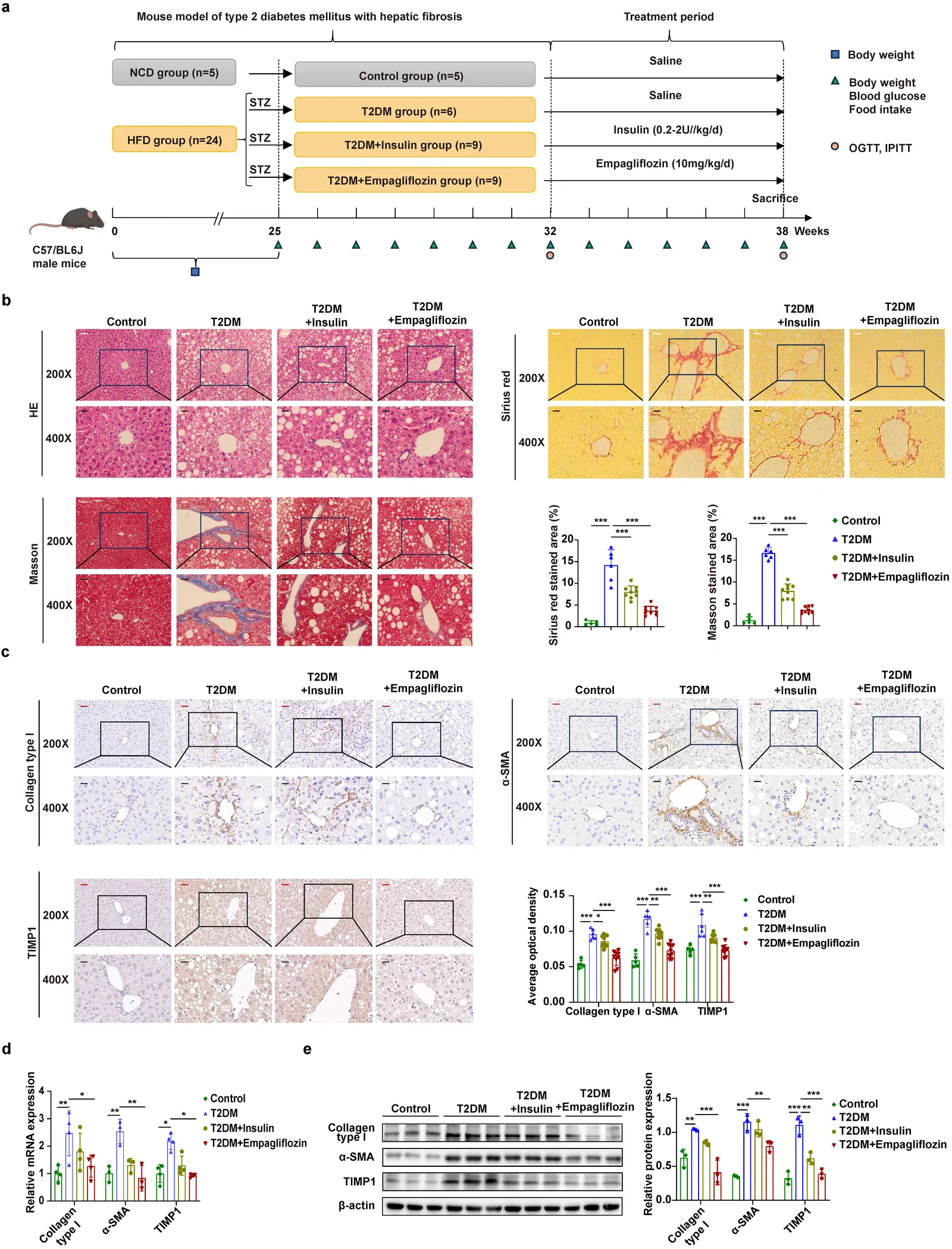
** Empagliflozin ameliorates HFD/STZ-induced hepatic fibrosis *in vivo.*** (a) Schematic diagram of the construction and intervention process for the T2DM with hepatic fibrosis mouse model (Created with Biorender.com). (b) Representative images of HE staining, Masson staining, and Sirius Red staining of liver tissue sections from mice in each group after 6 weeks of insulin or empagliflozin intervention. ImageJ software was used to perform quantitative analysis of the percentage of positive area in the Masson and Sirius red stained regions for each group. Scale bars: white, 50μm; black, 25μm. (c) Representative immunohistochemical images and quantitative statistical analysis of collagen type I, α-SMA, and TIMP1 in liver tissue sections from mice in each group after 6 weeks of insulin or empagliflozin intervention. Scale bars: white, 50μm; red, 25μm. (d) Gene expression of Collagen type I, α-SMA, and TIMP1 in liver tissue from mice in each group was measured using RT-qPCR. (e) Protein expression of collagen type I, α-SMA, and TIMP1 in liver tissue from mice in each group was measured by western blot, and protein quantification was performed using ImageJ software. Abbreviations: NCD, normal chow diet (n=5); HFD: high-fat diet (n=24); STZ, streptozotocin; T2DM, type 2 diabetes mellitus; Saline, physiological saline; OGTT, oral glucose tolerance test; IPITT, intraperitoneal insulin tolerance test. *P < 0.05, **P < 0.01, ***P < 0.001.

**Figure 2:**
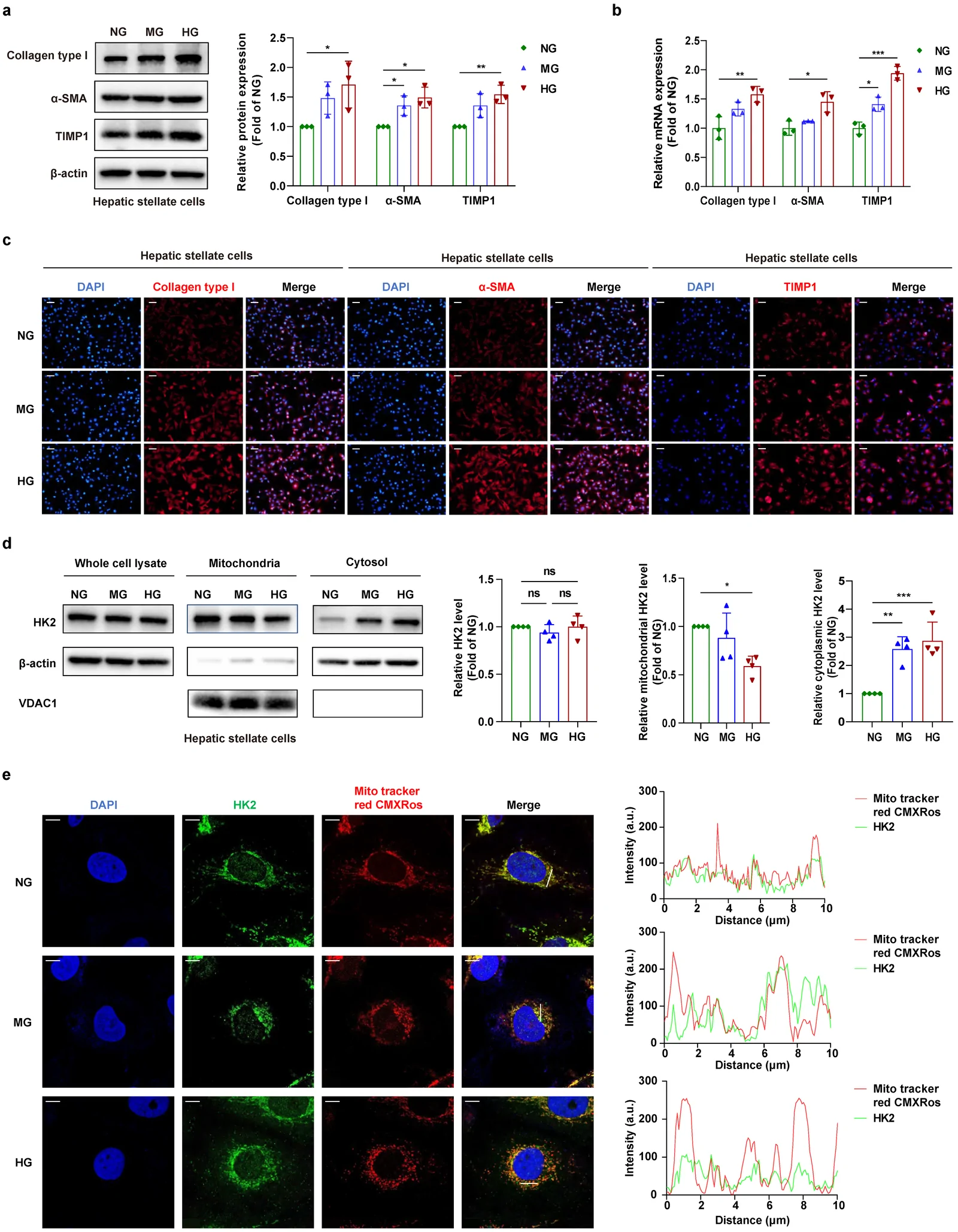
** High glucose promotes activation of HSCs and dissociation of HK2 from mitochondria *in vitro.*** (a) Western blot analysis of collagen type I, α-SMA, and TIMP1 protein expression in HSCs following NG, MG, and HG interventions. (b) RT-qPCR analysis of Collagen type I, α-SMA, and TIMP1 gene expression in HSCs after NG, MG, and HG interventions. (c) Immunofluorescence staining to assess Collagen type I, α-SMA, and TIMP1 protein expression in HSCs following NG, MG, and HG interventions. Scale bar: white, 100μm. (d) Western blot analysis of HK2 expression in whole-cell, mitochondrial, and cytoplasmic (removing mitochondria) fractions of HSCs after NG, MG, and HG interventions. (e) After immunofluorescence co-staining, confocal microscopy was used to observe changes in HK2 distribution in HSCs from NG, MG, and HG groups. MitoTracker Red CMXRos was used to label mitochondria (Red), and co-localization curves of HK2 (Green) and mitochondria (Red) were generated using ImageJ and GraphPad. Scale bar: white, 10μm. Abbreviations: NG, normal glucose; MG, moderate glucose; HG, high glucose. *P < 0.05, **P < 0.01, ***P < 0.001.

**Figure 3:**
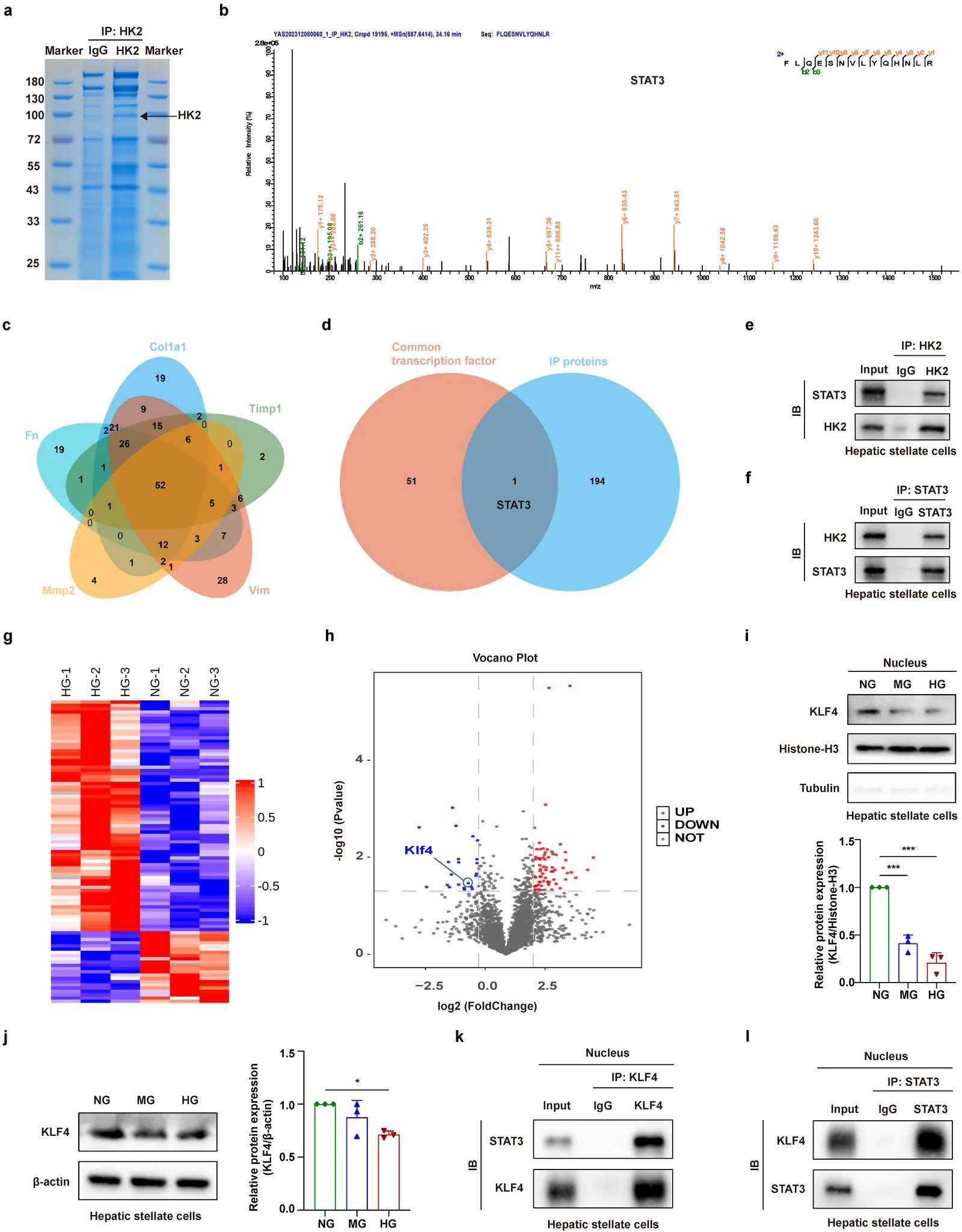
** Interaction between HK2 and STAT3 in the cytoplasm and STAT3 and KLF4 in the nucleus of HSCs.** (a) SDS-PAGE electrophoresis and Coomassie blue staining of proteins after immunoprecipitation with HK2 or IgG antibodies. (b) Representative MS plots and the corresponding peptide sequences derived from STAT3. (c) Venn diagram of common transcription factors predicted by HumanTFDB for HSC activation-related genes (including *Col1a1*, *Timp1*, *Fn*, *Mmp2*, and *Vim*). (d) Venn diagram showing the intersection between HK2-interacting proteins identified by Co-IP coupled with MS and common transcription factors predicted by HumanTFDB for HSC activation. Co-IP analysis of the interaction between HK2 and STAT3 in HSCs using HK2 (e) or STAT3 (f) as the capture antibody. Heatmap (g) and volcano plot (h) of nuclear proteomics analysis showing protein changes in the HSCs nucleus after intervention with different glucose concentrations (NG, HG). (i) Western blot analysis of nuclear KLF4 levels in HSCs after intervention with different glucose concentrations (NG, MG, HG). (j) Western blot analysis of whole-cell KLF4 levels in HSCs after intervention with different glucose concentrations (NG, MG, HG). Co-IP analysis of the interaction between KLF4 and STAT3 in the HSC nucleus using KLF4 (k) or STAT3 (l) as the capture antibody. Abbreviations: NG, normal glucose; MG, moderate glucose; HG, high glucose. *P < 0.05, **P < 0.01, ***P < 0.001.

**Figure 4:**
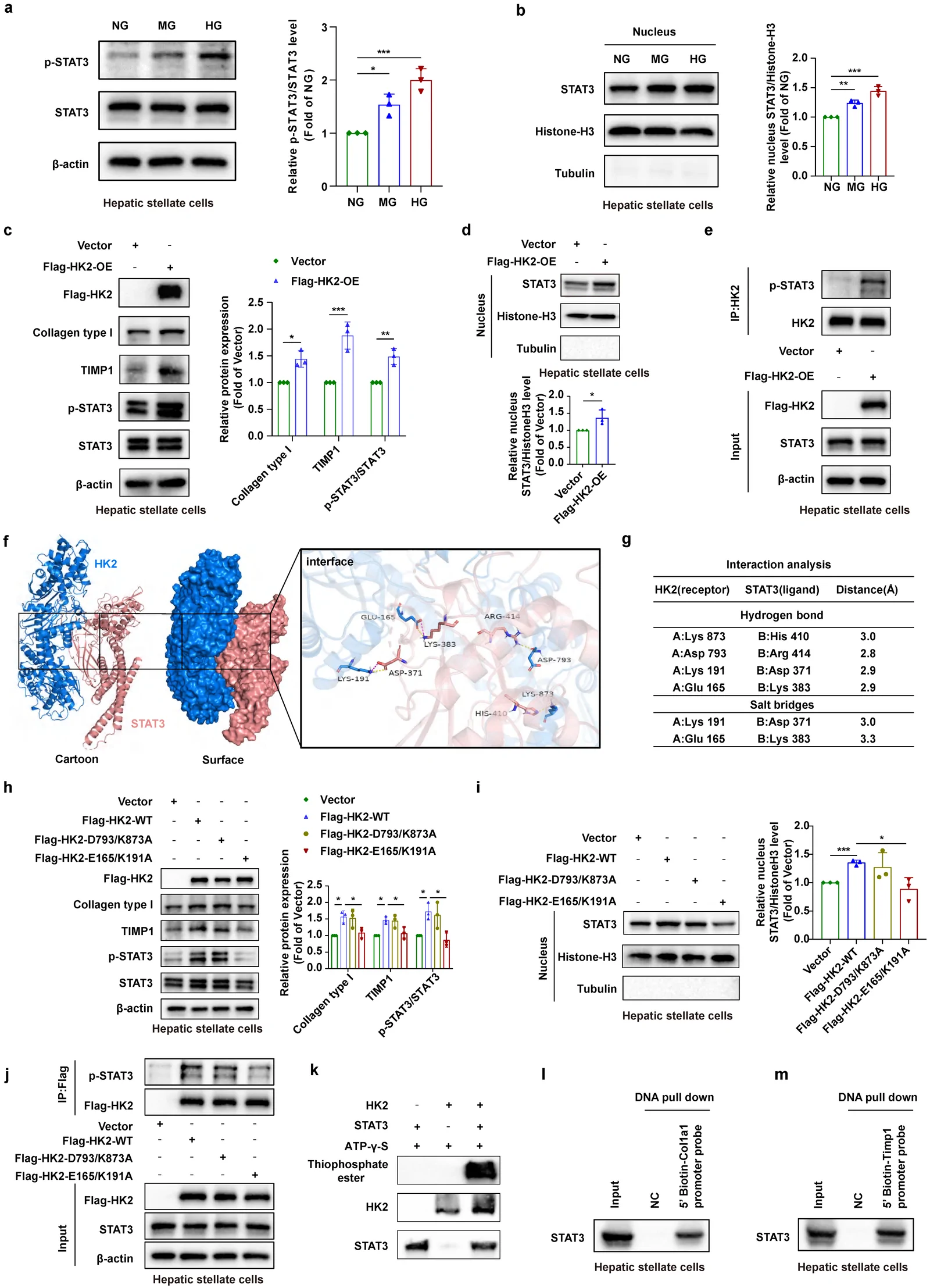
** Interaction between HK2 and STAT3 at the E165/K191 site facilitates STAT3 phosphorylation and nuclear translocation, regulating transcription of *Col1a1* and *Timp1.*** (a) Western blot analysis of the phosphorylation levels of STAT3 in HSCs after intervention with different glucose concentrations (NG, MG, HG). (b) Western blot analysis of STAT3 nuclear translocation in HSCs after intervention with different glucose concentrations (NG, MG, HG). (c) Western blot analysis and quantification of collagen type I, TIMP1, and phosphorylated STAT3 levels in HSCs transfected with Vector or Flag-HK2-OE. (d) Measurement of STAT3 nuclear translocation in HSCs transfected with Vector or Flag-HK2-OE. (e) Co-IP analysis of the level of phosphorylated STAT3 bound to HK2 in HSCs transfected with Vector or Flag-HK2-OE. (f) Molecular docking analysis on HK2 and STAT3 using Schrödinger software to predict their binding sites. (g) Predicted potential binding sites for HK2 and STAT3 based on Schrödinger software analysis. Based on these predictions, two HK2 mutant plasmids (Flag-HK2-D793/K873A and Flag-HK2-E165/K191A) were constructed, each containing a Flag tag and amino acid substitutions in the interaction domain. After transfection of HSCs with Vector, Flag-HK2-WT, Flag-HK2-D793/K873A, and Flag-HK2-E165/K191A, Western blot was performed to measure the levels of collagen type I, TIMP1, and phosphorylated STAT3 (h) and nuclear translocation of STAT3 (i). (j) Co-IP analysis of phosphorylated STAT3 bound to HK2 in HSCs transfected with Vector, Flag-HK2-WT, Flag-HK2-D793/K873A, and Flag-HK2-E165/K191A. (k) An *in vitro* kinase assay was performed using purified recombinant HK2 and STAT3 proteins to determine whether HK2 directly regulates STAT3 phosphorylation. DNA pull-down assays to verify the binding of STAT3 to the promoter regions of* Col1a1* (l) and *Timp1* (m) genes. Abbreviations: NG, normal glucose; MG, moderate glucose; HG, high glucose; NC, normal control. *P < 0.05, **P < 0.01, ***P < 0.001.

**Figure 5:**
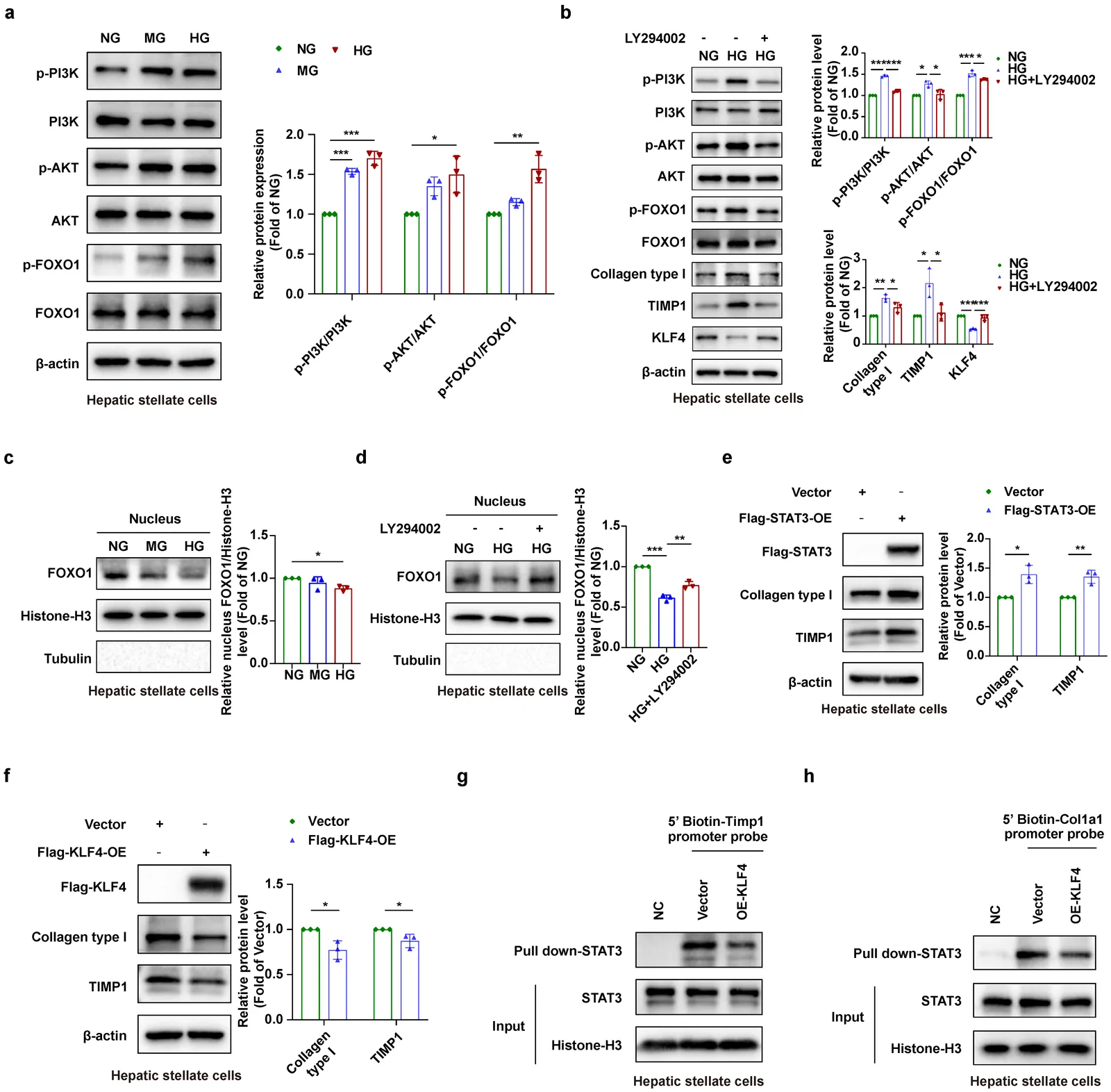
** High glucose downregulates the level of KLF4 in HSCs and reduces the inhibitory effect of KLF4 on STAT3 and DNA strand binding activity.** (a) Changes in p-PI3K/PI3K, p-AKT/AKT, p-FOXO1/FOXO1 in HSCs after intervention with different glucose concentrations (NG, MG, HG). (b) Changes in collagen type I, TIMP1, KLF4, and p-PI3K/PI3K, p-AKT/AKT, p-FOXO1/FOXO1 in HSCs after different treatments (NG, HG, HG+LY294002). (c) Nuclear translocation changes of FOXO1 in HSCs after intervention with different glucose concentrations (NG, MG, HG). (d) Nuclear translocation changes of FOXO1 in HSCs after different treatments (NG, HG, HG+LY294002). (e) Changes in collagen type I and TIMP1 detected by Western blot after transfection of Vector, Flag-STAT3-WT in HSCs. (f) Changes in collagen type I and TIMP1 detected by Western blot after transfection of Vector, Flag-KLF4-WT in HSCs. After transfection of Vector or Flag-KLF4-WT in HSCs, DNA pull-down was used to detect the changes in STAT3 binding to the promoter regions of the *Col1a1* (g) and *Timp1* (h) genes. Abbreviations: NG, normal glucose; MG, moderate glucose; HG, high glucose; NC, normal control; LY294002, PI3K inhibitor. *P < 0.05, **P < 0.01, ***P < 0.001.

**Figure 6:**
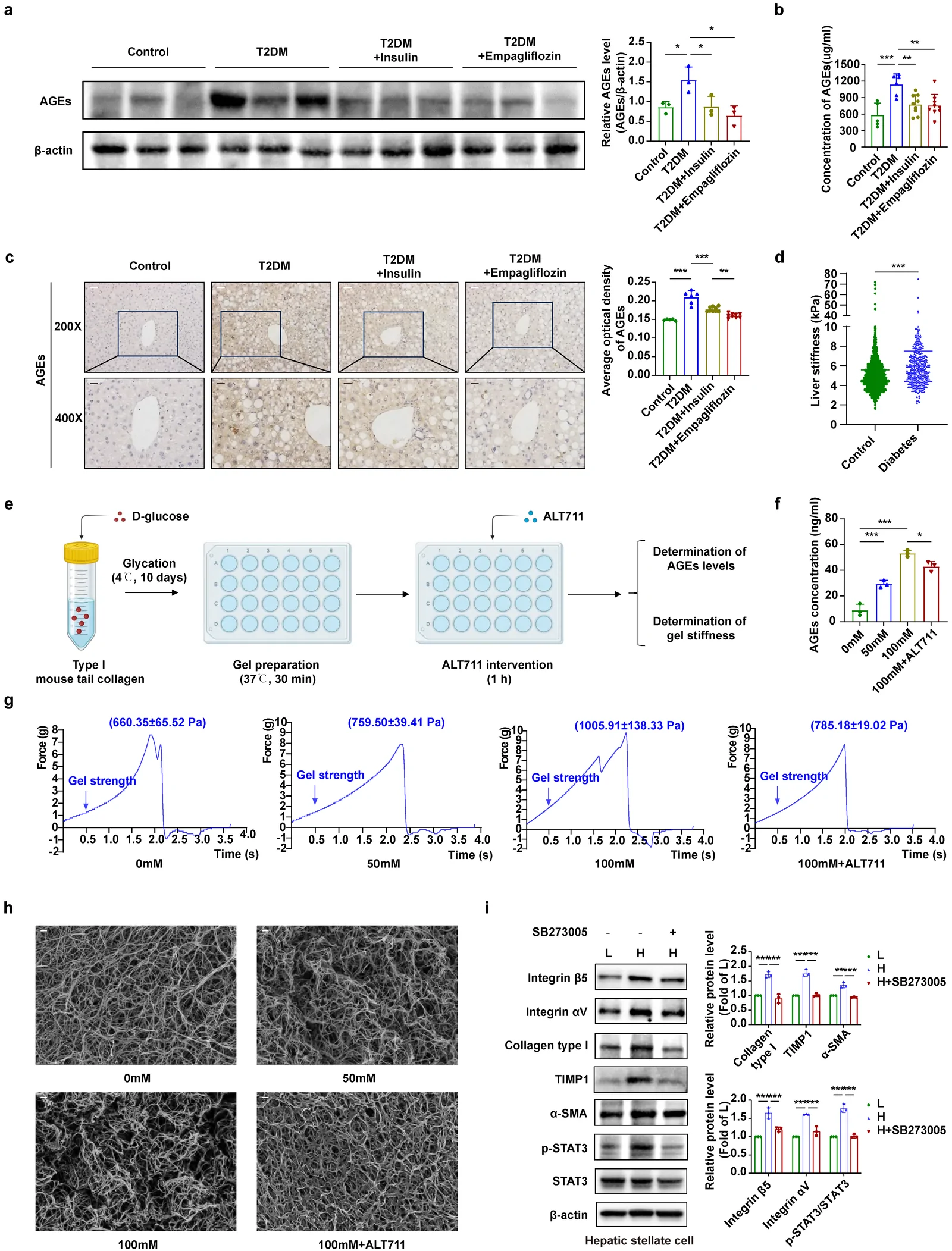
** Long-term high glucose promotes AGEs generation and increases ECM stiffness to enhance HSC activation.** Western blot (a) and ELISA (b) were used to detect the AGEs protein levels in the livers of mice from different groups (Control group, T2DM group, T2DM+Insulin group, T2DM+Empagliflozin group). (c) Representative immunohistochemistry images and quantitative analysis of AGEs in the liver of mice from different groups (Control group, T2DM group, T2DM+Insulin group, T2DM+Empagliflozin group). Scale bars: white, 50μm; black, 25μm. (d) Comparison of liver stiffness between diabetic (n=372) and non-diabetic (n=2355) populations using NHANES data. (e) Experimental flowchart for collagen glycation and ALT-711 intervention. (f) AGEs levels in type I mouse tail collagen gels after different treatments (0 mM, 50 mM, 100 mM, 100 mM + ALT711). (g) Force-time curves of type I mouse tail collagen gels from different groups (0 mM, 50 mM, 100 mM, 100 mM + ALT711) measured by a texture analyzer. (h) Scanning electron microscopy images showing changes in fiber arrangement in type I mouse tail collagen gels from different groups (0 mM, 50 mM, 100 mM, 100 mM + ALT711). Scale bar: white, 10μm. (i) Western blot analysis of changes in Integrin αV/β5, collagen type I, α-SMA, TIMP1, and pSTAT3/STAT3 in HSCs after different treatments (L, H, H+SB273005). Abbreviations: T2DM, type 2 diabetes; AGEs, advanced glycation end products; ALT711, AGE-breaker; SB273005, integrin αV/β5 inhibitor. *P < 0.05, **P < 0.01, ***P < 0.001.

**Figure 7:**
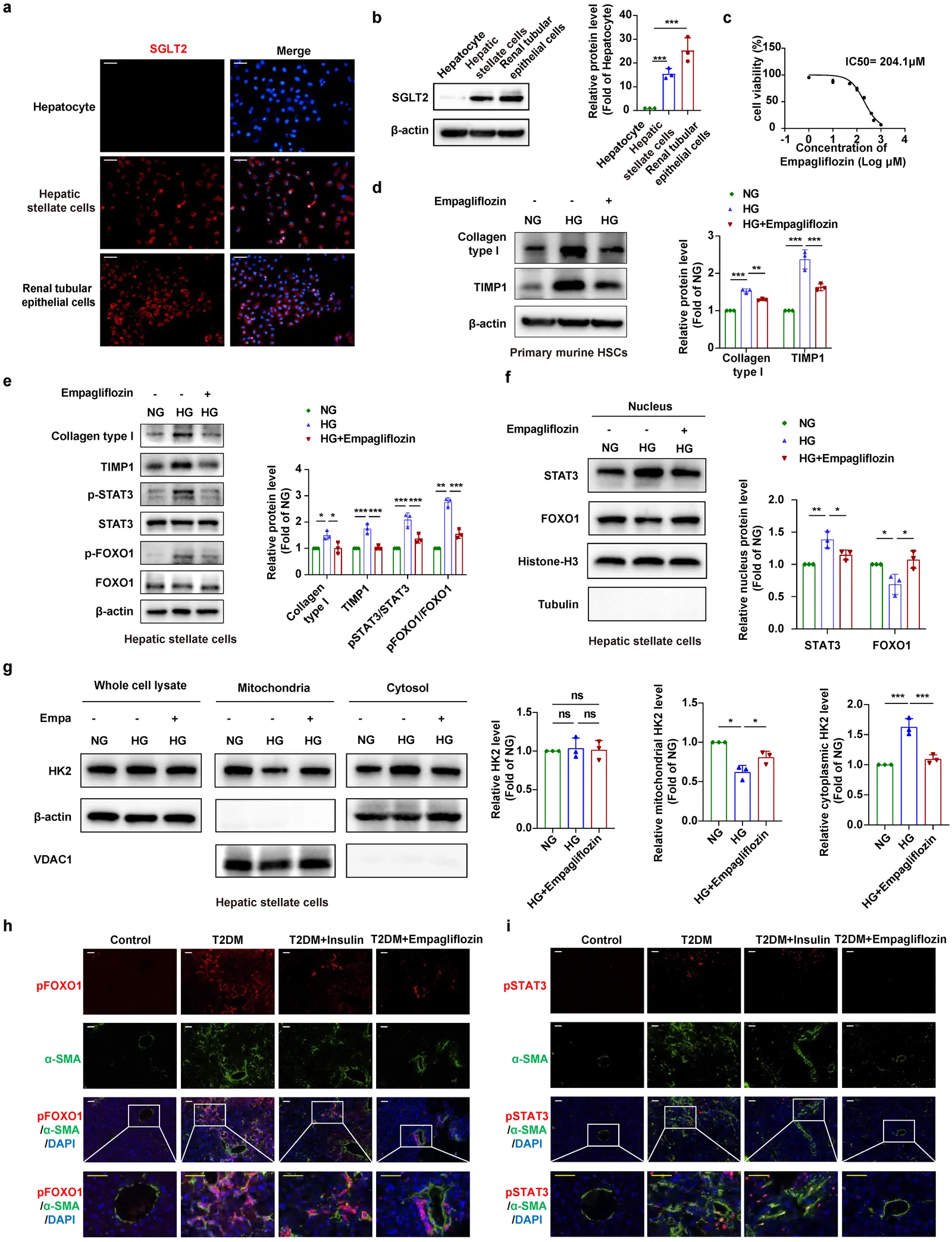
** Empagliflozin inhibits STAT3 and FOXO1 phosphorylation to suppress HSC activation.** Immunofluorescence (a) and western blot (b) were used to measure the SGLT2 protein expression in hepatocytes, hepatic stellate cells, and renal tubular epithelial cells. Scale bar: white, 50μm. (c) CCK8 assay was performed to assess the cell viability of HSCs after treatment with different concentrations of empagliflozin, and the IC50 of empagliflozin in HSCs was calculated. (d) Western blot analysis of changes in collagen type I, TIMP1 in primary murine HSCs after different treatments (NG, HG, HG+Empagliflozin). (e) Western blot analysis of changes in collagen type I, TIMP1, pSTAT3/STAT3, and pFOXO1/FOXO1 in HSCs after different treatments (NG, HG, HG+Empagliflozin). (f) Western blot analysis of nuclear translocation of STAT3 and FOXO1 in HSCs among different groups (NG, HG, HG+Empagliflozin). (g) Western blot analysis of changes in the distribution of HK2 in HSCs after different treatments (NG, HG, HG+Empagliflozin). (h) Representative immunofluorescence images of α-SMA and pFOXO1 co-staining in liver sections from mice of different groups (Control group, T2DM group, T2DM+Insulin group, T2DM+Empagliflozin group). (i) Representative immunofluorescence images of α-SMA and pSTAT3 co-staining in liver sections from mice of different groups (Control group, T2DM group, T2DM+Insulin group, T2DM+Empagliflozin group). Scale bars: white, 100μm; yellow, 100μm.

**Figure 8:**
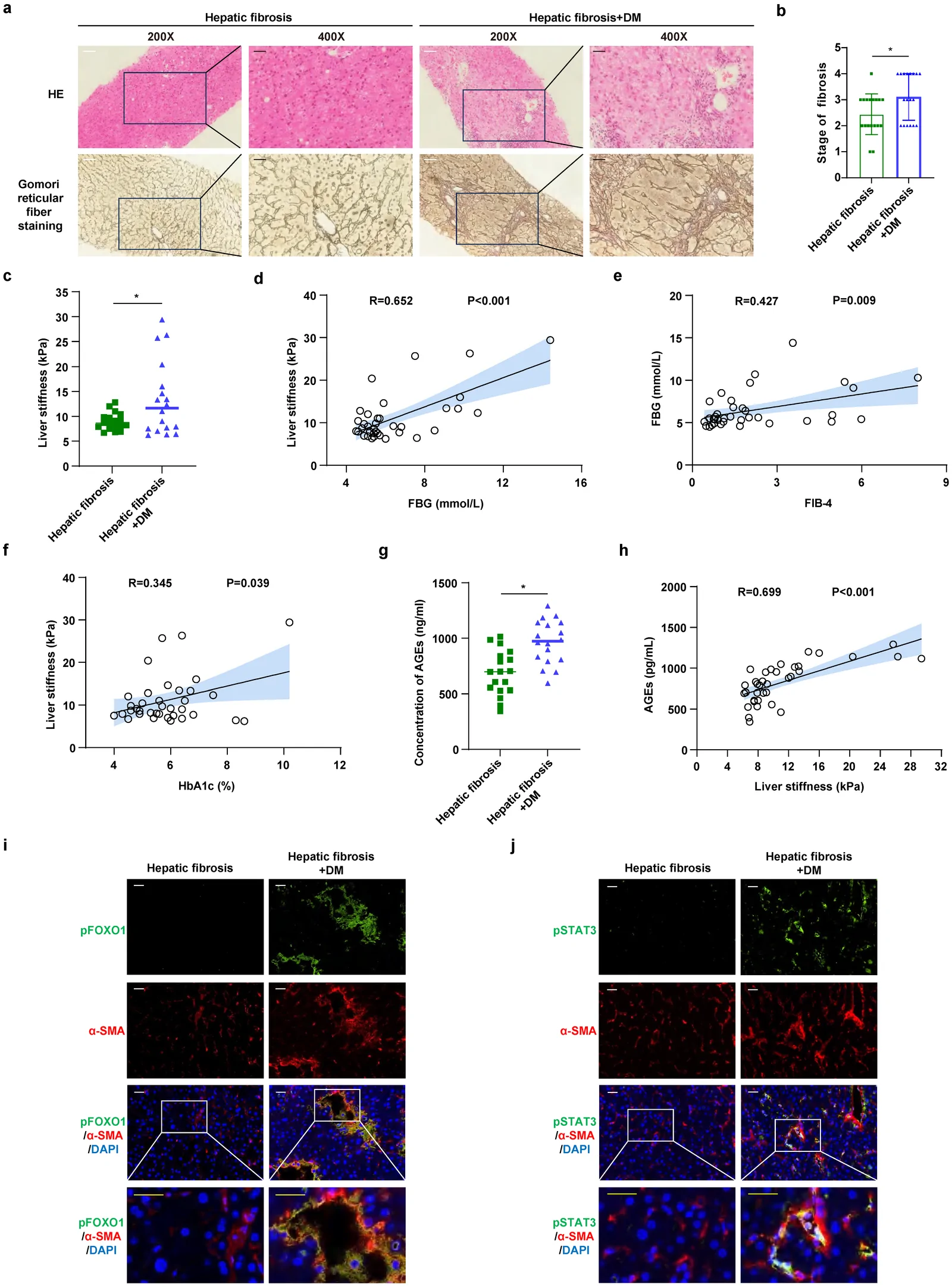
** Clinical significance of diabetes in hepatic fibrosis progression.** (a) Representative images of HE and Gomori reticular fiber staining of patients in the hepatic fibrosis group and the hepatic fibrosis + DM group. Scale bars: white, 50μm; black, 25μm. The stage of hepatic fibrosis (b) and liver stiffness levels (c) of patients in the hepatic fibrosis group and the hepatic fibrosis + DM group. Correlation analysis between FBG and liver stiffness (d) or FIB-4 (e) in patients with hepatic fibrosis. Correlation analysis between HbA1c and liver stiffness (f) in patients with hepatic fibrosis. Serum AGEs levels (g) in patients with hepatic fibrosis and their correlation with liver stiffness (h). (i) Representative immunofluorescence images of α-SMA and pFOXO1 co-staining in liver sections from human of different groups. (j) Representative immunofluorescence images of α-SMA and pSTAT3 co-staining in liver sections from human of different groups. Scale bars: white, 100μm; yellow, 100μm.

**Table 1:** Clinicopathological characteristics of patients with hepatic fibrosis.

Characteristics	Hepatic fibrosis (n = 18)	Hepatic fibrosis + DM (n = 18)	*P* value
Age, n (%)			0.257
≤ 59	16 (88.9%)	14 (77.8%)	
> 59	2 (11.1%)	4 (22.2%)	
Gender, n (%)			0.637
Male	8 (44.4%)	9 (50%)	
Female	10 (55.6%)	9 (50%)	
ALT (U/L), n (%)			0.629
≤ 40	10 (55.6%)	11 (61.1%)	
> 40	8 (44.4%)	7 (38.9%)	
AST (U/L), n (%)			0.155
≤ 35	11 (61.1%)	8 (44.4%)	
> 35	7 (38.9%)	10 (55.6%)	
ALB (g/L), n (%)			0.157
≤ 40	6 (33.3%)	9 (50%)	
> 40	12 (66.7%)	9 (50%)	
TB (μmol/L), n (%)			0.221
≤ 17.1	15 (83.3%)	12 (66.7%)	
> 17.1	3 (16.7%)	6 (33.3%)	
PLT (*10^9/L), mean ± sd	230.611 ± 63.437	182.111 ± 90.973	0.080
FBG (mmol/L), mean ± sd	5.344 ± 0.893	7.617 ± 2.471	**0.001**
HbA1c (%), mean ± sd	5.067 ± 0.607	6.722 ± 1.203	**< 0.001**
FIB-4, mean ± sd	1.384 ± 1.080	2.932 ± 2.157	**0.012**
Liver stiffness (kPa), mean ± sd	8.956 ± 1.694	13.356 ± 7.253	**0.020**
Degree of liver fibrosis, mean ± sd	2.444 ± 0.782	3.111 ± 0.875	**0.023**

ALT: Alanine aminotransferase; AST: Aspartate aminotransferase; ALB: Albumin; TB: Total bilirubin; PLT: Platelet; FBG: Fast blood glucose; FIB-4: Fibrosis-4.

## Data Availability

All relevant data supporting the findings of this study are available within the article and its supplementary materials. Additional data can be obtained from the corresponding author upon reasonable request.
