# Supplementary material for: Hyperglycemia in Diabetes Drives Hepatic Fibrosis Through the Non-Enzymatic Function of HK2 and AGEs-mediated Matrix Stiffening
Source: Int J Biol Sci. 2026 Aug 24;22(14):7533–56. doi: 10.7150/ijbs.130351 (PMC13617653; doi:10.7150/ijbs.130351)
Supplement: Supplementary file 1 [file ijbsv22p7533s1.pdf]

Supplementary figure legends

Fig.S1 Changes in body weight (a) and random blood glucose (b) during the construction of the T2DM with hepatic fibrosis mouse model. Changes in random blood glucose (c), body weight (d), daily food intake (e), and cumulative food intake (f) in mice after empagliflozin intervention. Changes in OGTT (g: week 0; h: week 6) and IPITT (i: week 0; j: week 6) in different groups of mice before and after empagliflozin treatment.

Fig.S2 (a) Western blot analysis of changes in collagen type I, α-SMA and TIMP1 in HSCs after different treatments (NG, HG, HG+BAY-876). Quantification of α-SMA (b), Collagen type I (c), and TIMP1 (d) immunofluorescence images in HSCs treated with different glucose concentrations using ImageJ. (e) Schematic diagram of mass spectrometry analysis identifying potential HK2-binding proteins in the cytoplasm under high-glucose conditions (Created with Biorender.com). (f) Western blot analysis was performed to determine the changes in STAT3 phosphorylation levels after HG treatment for different durations (1 h, 12 h, 24 h, and 48 h). (g) DNA pull-down assays to verify the binding of FOXO1 to the promoter regions of *klf4* gene. (h) Stiffness of type I mouse tail collagen gels (0, 50, 100 mM glucose, and 100 mM + ALT711) measured by texture analyzer. (i) Western blot analysis of changes in collagen type I, TIMP1 in HSCs after different treatments (NG, HG, HG+Stattic). (j) Western blot analysis was performed to determine SGLT2 protein expression in primary murine HSCs, hepatocytes, and renal tubular epithelial cells. (k) A three-dimensional tissue-like model was established using Matrigel, followed by high glucose and empagliflozin treatment. Afterwards, western blot analysis was performed to detect HK2 expression in whole-cell, mitochondrial, and cytoplasmic (removing mitochondria) fractions.

Supplementary files

Supplementary Table S1 Primer sequences for qRT PCR of mouse liver.

Supplementary Table S2 Primer sequences for qRT PCR of HSCs.

Supplementary Table S3 Plasmid construction information.

Supplementary Table S4 Prediction of potential STAT3 binding sites in the promoter region of *Col1a1* using JASPAR.

Supplementary Table S5 Prediction of potential STAT3 binding sites in the promoter region of *Timp1* using JASPAR.

Supplementary Table S6 Prediction of potential FOXO1 binding sites in the promoter region of *Klf4* using JASPAR.

Supplementary Table S7 Design of DNA probe sequence.

Supplementary Table S8 Potential candidate proteins for HK2 binding in the cytoplasm of HSCs under high glucose conditions identified by CoIP combined with MS.

Supplementary Table S9 Common transcription factors of *Col1a1*, *Timp1*, *Fn*, *Mmp2*, and *Vim* predicted by HumanTFDB.

Supplementary Table S10 Differential proteins in HSCs treated with different glucose concentrations (NG, HG) identified by nuclear proteomic

Fig.S1

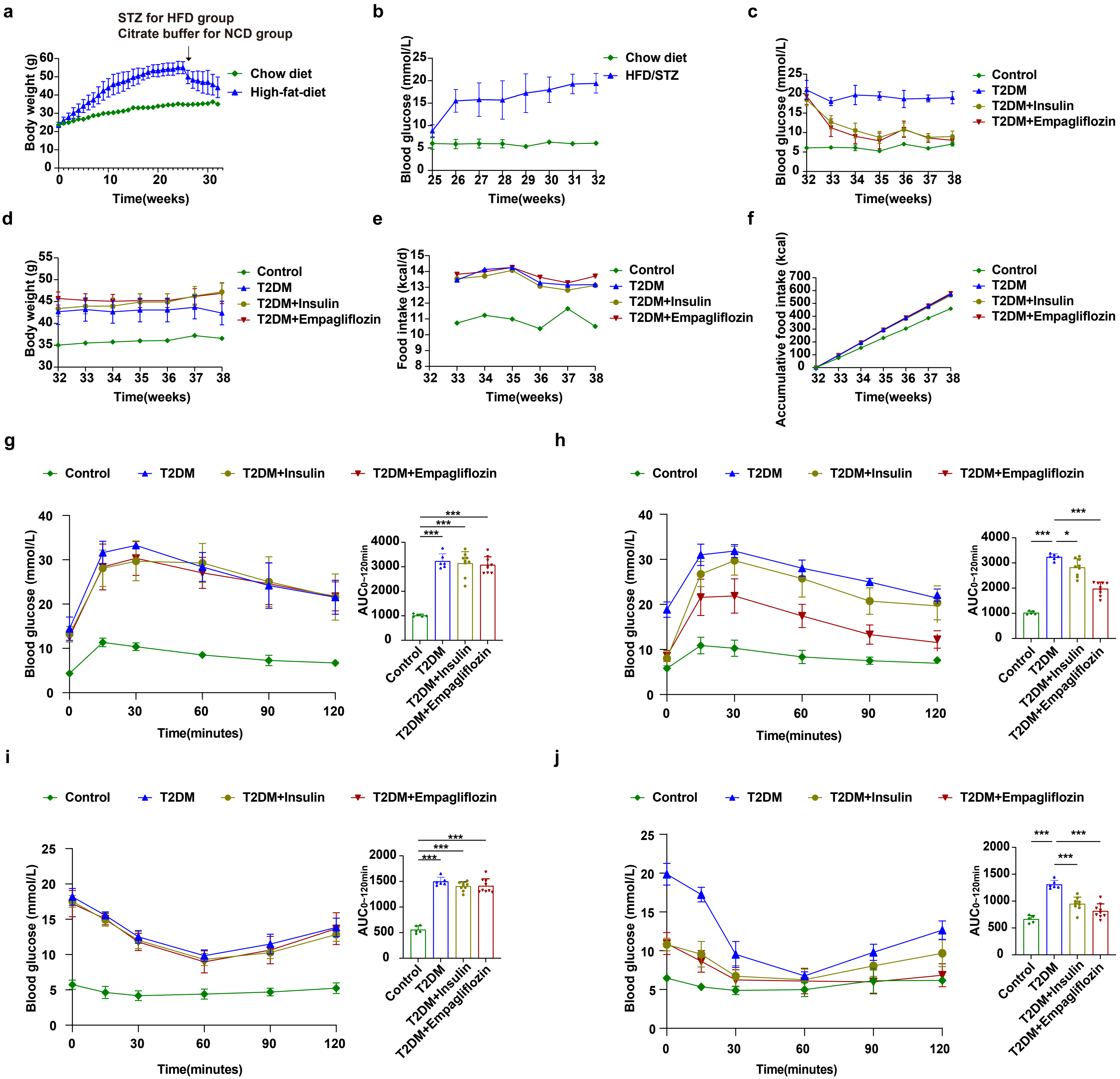

a
STZ for HFD group
Citrate buffer for NCD group
Body weight (g)
Time(weeks)
Chow diet
High-fat-diet
b
Blood glucose (mmol/L)
Time(weeks)
Chow diet
HFD/STZ
c
Blood glucose (mmol/L)
Time(weeks)
Control
T2DM
T2DM+Insulin
T2DM+Empagliflozin
d
Body weight (g)
Time(weeks)
e
Food intake (kcal/d)
Time(weeks)
f
Accumulative food intake (kcal)
Time(weeks)
g
Blood glucose (mmol/L)
Time(minutes)
AUC0~120min
h
i
j

Fig.S2

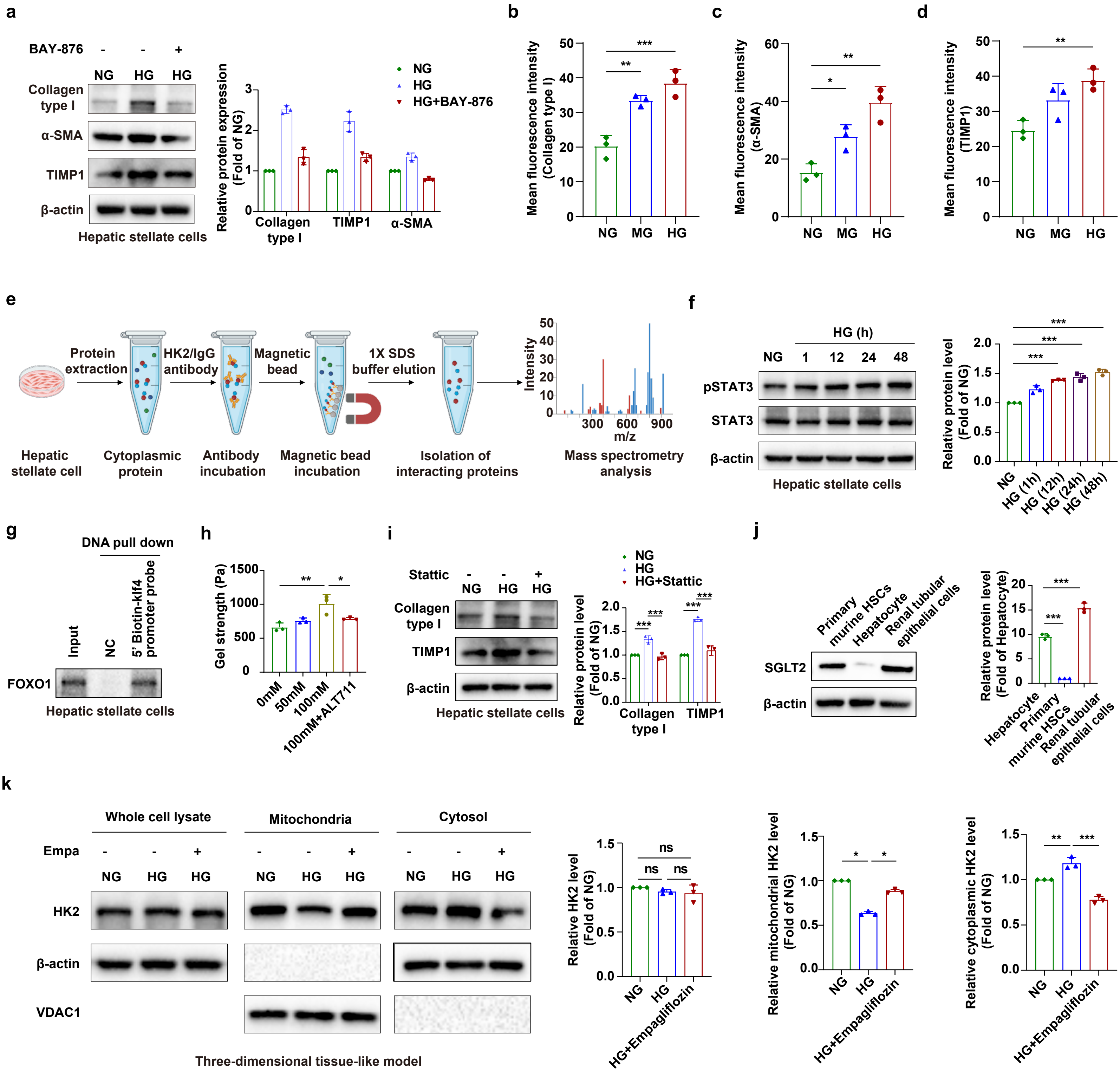
a
BAY-876
NG
HG
HG+BAY-876
Collagen type I
α-SMA
TIMP1
β-actin
Hepatic stellate cells
Relative protein expression (Fold of NG)
b
Mean fluorescence intensity (Collagen type I)
NG
MG
HG
c
Mean fluorescence intensity (α-SMA)
d
Mean fluorescence intensity (TIMP1)
e
Protein extraction
HK2/IgG antibody
Magnetic bead
1X SDS buffer elution
Hepatic stellate cell
Cytoplasmic protein
Antibody incubation
Magnetic bead incubation
Isolation of interacting proteins
Intensity
m/z
Mass spectrometry analysis
f
HG (h)
pSTAT3
STAT3
Relative protein level (Fold of NG)
g
DNA pull down
Input
NC
5' Biotin-klf4 promoter probe
FOXO1
h
Gel strength (Pa)
0mM
50mM
100mM
100mM+ALT711
i
Stattic
HG+Stattic
Relative protein level (Fold of NG)
j
Primary murine HSCs
Hepatocyte
Renal tubular epithelial cells
SGLT2
Relative protein level (Fold of Hepatocyte)
k
Whole cell lysate
Mitochondria
Cytosol
Empa
HK2
VDAC1
Three-dimensional tissue-like model
Relative HK2 level (Fold of NG)
Relative mitochondrial HK2 level (Fold of NG)
Relative cytoplasmic HK2 level (Fold of NG)
HG+Empagliflozin

Supplementary Table S1. Primer sequences for qRT PCR of mouse liver.

Gene name	Forward primer (5'-3')	Reverse primer (5'-3')
Col1a1	TAAGGGTCCCCAATGGTGAGA	GGGTCCCTCGACTCCTACAT
Acta2	CCCAGACATCAGGGAGTAATGG	TCTATCGGATACTTCAGCGTCA
Timp1	GCAACTCGGACCTGGTCATAA	CGGCCCGTGATGAGAAACT
Actb	GTGACGTTGACATCCGTAAAGA	GCCGGACTCATCGTACTCC

Supplementary Table S2. Primer sequences for qRT PCR of HSCs.

Gene name	Forward primer (5'-3')	Reverse primer (5'-3')
Col1a1	GAGGGCCAAGACGAAGACATC	CAGATCACGTCATCGCACAAC
Acta2	GAGCGTGGCTATTCCTTCGT	TTCAAAGTCCAGAGCTACATAACACAGT
Timp1	CTTCTGCAATTCCGACCTCGT	ACGCTGGTATAAGGTGGTCTG
Actb	GCCTCGCCTTTGCCGATCC	GCGCGGCGATATCATCATCCA

Supplementary Table S3. Plasmid construction information.

Gene name	Species	Plasmid vector	PCR amplicon size
HK2（NM_000189.5）	Human	GV657	2797
HK2（NM_000189.5（D793/K873A））	Human	GV657	2800
HK2（NM_000189.5（E165/K191A））	Human	GV657	2800
STAT3（NM_139276.3)	Human	GV657	2356
KLF4（NM_004235.6）	Human	GV657	1486

Supplementary Table S4. Prediction of potential STAT3 binding sites in the promoter region of *Col1a1* using JASPAR.

Matrix ID	Name	Score	Relative score	Sequence ID	Start	End	Strand	Predicted sequence
MA0144.3	MA0144.3.STAT3	13.89	0.98	COL1A1	972	980	+	TGCTGGGAA
MA0144.3	MA0144.3.STAT3	11.58	0.95	COL1A1	741	749	-	TTCTTGAAA
MA0144.3	MA0144.3.STAT3	9.08	0.91	COL1A1	1485	1493	-	AGCTGGGAA
MA0144.3	MA0144.3.STAT3	7.59	0.90	COL1A1	1681	1689	+	TCCCAGGAA
MA0144.3	MA0144.3.STAT3	6.88	0.89	COL1A1	742	750	+	TTCAAGAAA
MA0144.3	MA0144.3.STAT3	6.31	0.88	COL1A1	551	559	-	TCCTGAGAA
MA0144.3	MA0144.3.STAT3	6.06	0.88	COL1A1	907	915	-	TTCTGGGAG
MA0144.3	MA0144.3.STAT3	5.29	0.87	COL1A1	5	13	+	CTCTTGGAA

Supplementary Table S5. Prediction of potential STAT3 binding sites in the promoter region of *Timp1* using JASPAR.

Matrix ID	Name	Score	Relative score	Sequence ID	Start	End	Strand	Predicted sequence
MA0144.3	MA0144.3.STAT3	8.09	0.90	TIMP1	1910	1918	+	ATCCAGGAA
MA0144.3	MA0144.3.STAT3	6.92	0.89	TIMP1	68	76	+	TGCCTGTAA
MA0144.3	MA0144.3.STAT3	5.19	0.86	TIMP1	1102	1110	-	AGCAGGGAA

Supplementary Table S6. Prediction of potential FOXO1 binding sites in the promoter region of *Klf4* using JASPAR.

Matrix ID	Name	Score	Relative score	Sequence ID	Start	End	Strand	Predicted sequence
MA0480.2	FOXO1	13.25	0.99	KLF4	279	289	+	AAGTAAACACC
MA0480.2	FOXO1	8.80	0.87	KLF4	734	744	-	AATTAAACACT
MA0480.2	FOXO1	8.46	0.87	KLF4	350	360	-	TAGCAAACAGT
MA0480.2	FOXO1	7.80	0.85	KLF4	453	463	+	AAGTAAAGATC
MA0480.1	FOXO1	7.43	0.86	KLF4	1856	1866	+	GTTTGTTGATT
MA0480.2	FOXO1	4.79	0.77	KLF4	513	523	-	AAGTGCACACC
MA0480.2	FOXO1	4.79	0.77	KLF4	1223	1233	-	CTTTAAATAGC
MA0480.1	FOXO1	4.79	0.81	KLF4	1108	1118	-	TCCTTTTTTTT
MA0480.2	FOXO1	4.68	0.77	KLF4	67	77	+	GTTCAAACAGG
MA0480.3	FOXO1	4.68	0.78	KLF4	897	903	+	GCCAACA

Supplementary Table S7. Design of DNA probe sequence.

Primer name	Sequence (5' to 3')	Number of bases	5' end modification
Biotin-COL1A1 promoter	ATGCAGCTCCCAGAAAGACACCC CTTTAGCTAAGTGCCCTCCCTCCA CCCAGGTTCTCTCTGGTTTGACTG TGCTGGGAAGGAGGGTCTC	90	5'Biotin
COL1A1 promoter	ATGCAGCTCCCAGAAAGACACCC CTTTAGCTAAGTGCCCTCCCTCCA CCCAGGTTCTCTCTGGTTTGACTG TGCTGGGAAGGAGGGTCTC	90	-
Biotin-TIMP1 promoter	GAGTAATGCATCCAGGAAGCCTGG AGGCCTGTGGTTTCCGCACCCGC TGCCACCCCCGCCCCTAGCGTGG ACATTTATCCTCTAGCGCTC	90	5'Biotin
TIMP1 promoter	GAGTAATGCATCCAGGAAGCCTGG AGGCCTGTGGTTTCCGCACCCGC TGCCACCCCCGCCCCTAGCGTGG ACATTTATCCTCTAGCGCTC	90	-
Biotin-KLF4 promoter	ATCCAAAGTAAACACCTCGCCAAA CCTACGTTTAGAAAGTGATACCGA ACACCACATTCACGTTTTAAAAAAA AATACTGTTTGCTAAGA	90	5'Biotin
KLF4 promoter	ATCCAAAGTAAACACCTCGCCAAA CCTACGTTTAGAAAGTGATACCGA ACACCACATTCACGTTTTAAAAAAA AATACTGTTTGCTAAGA	90	-

Supplementary Table S8 Potential candidate proteins for HK2 binding in the cytoplasm of HSCs under high glucose conditions identified by CoIP combined with MS.

Protein names	Gene names
Septin-7	SEPT7;DKFZp686F17268;Nbla02942;LOC392884;DKFZp586I031
Programmed cell death protein 6	PDCD6
EF-hand domain-containing protein D2;EF-hand domain-containing protein D1	EFHD2;EFHD1;PP1187;FLJ13612;MST133
Ataxin-2-like protein	ATXN2L
Coiled-coil domain-containing protein 6	CCDC6
Protein pelota homolog	hCG_2002731;PELO
AP-3 complex subunit mu-1;AP-3 complex subunit mu-2	DKFZp586G1518;AP3M1;AP3M2
Disabled homolog 2	DAB2
S-phase kinase-associated protein 2	SKP2
Nuclear pore complex protein Nup155	NUP155
Adenosylhomocysteinase;Putative adenosylhomocysteinase 2;Putative adenosylhomocysteinase 3	AHCYL1;AHCYL2;FGFR2-AHCYL1
RelA-associated inhibitor	PPP1R13L
Splicing factor 3A subunit 1	SF3A1
ATP-citrate synthase	ACLY;ACLY variant protein
Synaptic vesicle membrane protein VAT-1 homolog	VAT1
cAMP-dependent protein kinase type II-alpha regulatory subunit	PRKAR2A
Elongation factor 1-beta	EEF1B2;LOC392793
Small ubiquitin-related modifier;Small ubiquitin-related modifier 1	SUMO1
TAR DNA-binding protein 43	TARDBP;TDP43
Tubulin--tyrosine ligase-like protein 12	TTLL12
Neutral alpha-glucosidase AB	GANAB;HEL-S-164nA
Ras-related protein Rab-6B;Ras-related protein Rab-6A;Ras-related protein Rab-39A	RAB6B;RAB6A;RAB39A
Protein phosphatase Slingshot homolog 3	SSH3
	NEDD4
Maspardin	SPG21
TBC1 domain family member 1;TBC1 domain family member 4	TBC1D1;TBC1D4
Heat shock-related 70 kDa protein 2	HSPA2
Eukaryotic translation initiation factor 5	EIF5
Nicotinamide phosphoribosyltransferase	NAMPT;PBEF1
Nucleus accumbens-associated protein 1	NACC1;BTBD14B
Transportin-2	TNPO2;TNPO2 variant protein
AP-1 complex subunit mu-1	AP1M1
Delta-aminolevulinic acid dehydratase	ALAD
60 kDa SS-A/Ro ribonucleoprotein	TROVE2
Transmembrane protein 74	TMEM74
Mitogen-activated protein kinase kinase kinase 2;Mitogen-activated protein kinase kinase kinase 3	MAP3K2;MAP3K3

Myosin light polypeptide 6;Myosin light chain 6B	MYL6;MYL6B
TRAF-type zinc finger domain-containing protein 1	TRAFD1
Nucleosome assembly protein 1-like 4	NAP1L4
Guanine nucleotide-binding protein-like 1	HSR1;GNL1
Ras GTPase-activating protein-binding protein 2	G3BP2
Focal adhesion kinase 1	PTK2;FAK;DKFZp666O0110
40S ribosomal protein S19	RPS19
GRIP1-associated protein 1	GRIPAP1
Clathrin heavy chain;Clathrin heavy chain 1	CLTC
DNA-directed RNA polymerases I, II, and III subunit RPABC1	POLR2E
Guanine nucleotide-binding protein G(t) subunit alpha-2	GNAT2
Kinesin-like protein;Kinesin-like protein KIF3A	KIF3A
Serine/arginine-rich splicing factor 3	SRSF3;SFRS3
Proteasome subunit beta type;Proteasome subunit beta type-6	PSMB6
NEDD8 ultimate buster 1	NYREN18;NUB1
GTP-binding protein Rheb	RHEB
Zinc finger protein 638	ZNF638;NP220
Protein FAM227A	FAM227A
Protein inscuteable homolog	INSC
Disks large homolog 1	DLG1
Cullin-associated NEDD8-dissociated protein 1	CAND1
Matrin-3	DKFZp686L22104;DKFZp686K0542;MATR3;DKFZp686K23100
Clathrin interactor 1	CLINT1
RNA-binding protein 39	RBM39;DKFZp686A11192;DKFZp686C17209;RNPC2;DKFZp781C0423
S-formylglutathione hydrolase	ESD
Serine/threonine-protein phosphatase;Serine/threonine-protein phosphatase 2A catalytic subunit beta isoform	PPP2CB
Protein phosphatase 1 regulatory subunit 7	PPP1R7
A-kinase anchor protein 8-like	AKAP8L
Ras-related protein Rab-7a	RAB7A
Protein unc-45 homolog A	UNC45A
Survival motor neuron protein	SMN2;SMN1
Phosphatidylinositol 3,4,5-trisphosphate 3-phosphatase TPTE2	TPTE2
Band 4.1-like protein 2	EPB41L2
Dipeptidyl peptidase 9	DPP9
Transcription elongation factor B polypeptide 2	TCEB2
Heterogeneous nuclear ribonucleoprotein H2	HNRNPH2
Vacuolar protein sorting-associated protein 29	DKFZp667O202;VPS29
EVI5-like protein	EVI5L
Septin-2	2-Sep
Lamin-B2	LMNB2
Far upstream element-binding protein 2	KHSRP
Protein phosphatase 1B	PPM1B
Transforming acidic coiled-coil-containing protein 3	TACC3

Annexin A1;Annexin	ANXA1
Zinc finger CCCH-type antiviral protein 1	ZC3HAV1
Signal transducer and activator of transcription;Signal transducer and activator of transcription 3	STAT3
Eukaryotic translation initiation factor 4H	WBSCR1;LOC392647;EIF4H
Signal recognition particle 54 kDa protein	SRP54
Probable ATP-dependent RNA helicase DDX58	DDX58
Solute carrier family 13 member 4	SLC13A4
Myosin light chain kinase, smooth muscle;Myosin light chain kinase, smooth muscle, deglutamylated form	MYLK
Elongation factor Tu GTP-binding domain-containing protein 1	EFTUD1
DNA-directed RNA polymerase III subunit RPC6	POLR3F
Transferrin receptor protein 1;Transferrin receptor protein 1, serum form	TFRC
WD repeat-containing protein 1	WDR1;HEL-S-52
26S proteasome non-ATPase regulatory subunit 8	PSMD8;HEL-S-91n
Formin-binding protein 1	FNBP1
Nucleoporin Nup43	NUP43
40S ribosomal protein S6	RPS6
UPF0600 protein C5orf51	C5orf51
DNA polymerase;DNA polymerase alpha catalytic subunit	POLA1
Cyclin-dependent kinase inhibitor 2A	CDKN2A;p14ARF
Cdc42-interacting protein 4	TRIP10;STP
Vacuolar protein sorting-associated protein 26A	VPS26A
Replication factor C subunit 5	RFC5
Methionine--tRNA ligase, cytoplasmic	MARS
Disks large-associated protein 5	DLGAP5
Serine/threonine-protein phosphatase 4 regulatory subunit 1	PPP4R1
Nucleolar transcription factor 1	UBTF
Thymidylate synthase	TYMS
FAS-associated factor 1	FAF1
Ras suppressor protein 1	RSU1
26S proteasome non-ATPase regulatory subunit 10	PSMD10
U1 small nuclear ribonucleoprotein A	SNRPA
Breast cancer anti-estrogen resistance protein 1	BCAR1
Proteasome assembly chaperone 1	PSMG1
Sorting nexin-15	SNX15;hCG_2044837
SEC23-interacting protein	SEC23IP
Condensin complex subunit 1	NCAPD2
Protein HEXIM1	HEXIM1
T-complex protein 11-like protein 1	TCP11L1
Histone deacetylase;Histone deacetylase 1;Histone deacetylase 2	HDAC1;HDAC2
General transcription factor IIF subunit 1	GTF2F1
Pyridoxal-dependent decarboxylase domain-containing protein 1	PDXDC1
Pantothenate kinase 4	PANK4
Protein flightless-1 homolog	FLII
Obg-like ATPase 1	OLA1;PTD004

rRNA 2-O-methyltransferase fibrillarin	FBL
Rab GDP dissociation inhibitor beta	GDI2
Protein PRRC2C	PRRC2C
BSD domain-containing protein 1	BSDC1
Asparagine--tRNA ligase, cytoplasmic	NARS
Ribonucleoside-diphosphate reductase;Ribonucleoside-diphosphate reductase large subunit	RRM1
Annexin;Annexin A11	ANXA11
Ras-related GTP-binding protein C;Ras-related GTP-binding protein D	RRAGC;RRAGD
G1/S-specific cyclin-D1	CCND1;cyclin D1
26S proteasome non-ATPase regulatory subunit 11	PSMD11
Eukaryotic translation initiation factor 3 subunit K	EIF3K
Interferon-induced protein 44	IFI44
Calcium-binding and coiled-coil domain-containing protein 2	CALCOCO2
Tyrosine-protein kinase receptor;Sequestosome-1	SQSTM1;SQSTM1-ALK
Eukaryotic translation initiation factor 2 subunit 2	EIF2S2
Protein NDRG3	NDRG3
WD repeat-containing protein 44	WDR44;DKFZp761M142
Alpha-parvin	PARVA
RNA-binding protein 12B	RBM12B
General transcription factor IIE subunit 1	GTF2E1
AP-1 complex subunit beta-1	AP1B1
Eukaryotic translation initiation factor 5A;Eukaryotic translation initiation factor 5A-2;Eukaryotic translation initiation factor 5A-1-like;Eukaryotic translation initiation factor 5A-1	EIF5A2;EIF5A;EIF5AL1
COP9 signalosome complex subunit 3	COPS3
Transient receptor potential cation channel subfamily M member 2	TRPM2
Ubiquitin carboxyl-terminal hydrolase;Probable ubiquitin carboxyl-terminal hydrolase FAF-X	USP9X
Nucleolysin TIA-1 isoform p40	TIA1
Phospholipase A-2-activating protein	PLAA
Eukaryotic initiation factor 4A-II;Eukaryotic initiation factor 4A-II, N-terminally processed	EIF4A2
Translation initiation factor eIF-2B subunit delta	DKFZp586J0119;EIF2B4
Poly(rC)-binding protein 3	PCBP3
Thyroid receptor-interacting protein 6	TRIP6i1;TRIP6
Keratin, type II cytoskeletal 74;Keratin, type II cytoskeletal 73;Keratin, type II cytoskeletal 72;Keratin, type II cytoskeletal 71	KRT74;KRT73;KRT72;KRT71
Large subunit GTPase 1 homolog	LSG1
Sister chromatid cohesion protein PDS5 homolog A	PDS5A
LYR motif-containing protein 5	LYRM5
	TUBB3
Translation initiation factor eIF-2B subunit gamma	EIF2B3
Fibroblast growth factor	FGF7
Neuralized-like protein 4	NEURL4
Gem-associated protein 4	GEMIN4
Small nuclear ribonucleoprotein Sm D1	SNRPD1

YTH domain-containing protein 1	YTHDC1
Myosin regulatory light chain 12A;Myosin regulatory light chain 12B	MYL12A;MYL12B
Small nuclear ribonucleoprotein Sm D2	SNRPD2
Histone acetyltransferase type B catalytic subunit	HAT1
U3 small nucleolar RNA-interacting protein 2	RRP9
DnaJ homolog subfamily C member 8	DNAJC8
T-complex protein 1 subunit alpha	TCP1
Tubulin alpha-4A chain	TUBA4A
Prolyl 3-hydroxylase 1	LEPRE1;P3H1
Reticulon;Reticulon-4	RTN4
E3 ubiquitin-protein ligase ARIH1	ARIH1
Vacuolar protein sorting-associated protein 16 homolog	VPS16
	DKFZp762B162
Condensin complex subunit 3	NCAPG
POTE ankyrin domain family member E;POTE ankyrin domain family member I;POTE ankyrin domain family member J;Putative beta-actin-like protein 3;Putative beta-actin-like protein 3, N-terminally processed	POTEE;POTEI;POTEJ;POTEKP;POTEM
Ras-related GTP-binding protein A;Ras-related GTP-binding protein B	RRAGA;RRAGB
Golgi to ER traffic protein 4 homolog	GET4
Histone-arginine methyltransferase CARM1	CARM1
Peptidase M20 domain-containing protein 2	PM20D2
Kinesin-associated protein 3	KIFAP3
Protein fem-1 homolog C	FEM1C
Protein PRRC1	PRRC1
Arf-GAP with Rho-GAP domain, ANK repeat and PH domain-containing protein 1	ARAP1
Importin-9	IPO9
Membrane-spanning 4-domains subfamily A member 10	MS4A10
RUN and FYVE domain-containing protein 1	RUFY1
PITH domain-containing protein 1	PITHD1
Kinesin light chain 2	KLC2
Sorting nexin-7	SNX7
Serine/threonine-protein phosphatase 6 regulatory subunit 1	PPP6R1

Supplementary Table S9 Common transcription factors of *Col1a1*, *Timp1*, *Fn*, *Mmp2*, and *Vim* predicted by HumanTFDB.

Common transcription factors
SPI1
BRD4
CEBPB
E2F1
EP300
ERG
MAZ
MED1
RUNX1
STAT1
ETS1
EGR1
FOS
FOSL1
JUN
JUND
KDM5B
MAX
MYC
NR2F2
POLR2A
RCOR1
TEAD4
USF1
YY1
FOSL2
GATA3
NFIC
REST
SIN3A
TAF1
TCF12
AR
CTCF
FOXA1
RAD21
STAG1
STAT3
TFAP2C
FOXA2
SP1
GATA4
KLF4
ELF1
SMAD3

CHD1
LMNB1
FLI1
MYOD1
KDM4C
KLF9
MYH11

Supplementary Table S10 Differential proteins in HSCs treated with different glucose concentrations (NG, HG) identified by nuclear proteomics.

Protein Name	Gene Name	HG/NG	t test p value
Glycogenin-1	GYG1	9.12887	0.010176
Glypican-1	GPC1	7.883804	0.018215
Protein HEG homolog 1	HEG1	5.848456	0.026628
Probable U3 small nucleolar RNA-associated protein 11	UTP11	5.225786	0.020467
NPC intracellular cholesterol transporter 2	NPC2	5.047073	2.93E-06
Trophoblast glycoprotein	TPBG	4.717855	0.021238
WD repeat-containing protein 46	WDR46	4.540654	0.011445
Heparanase	HPSE	4.516952	0.028766
39S ribosomal protein L17, mitochondrial	MRPL17	4.500567	0.00788
Surfeit locus protein 6	SURF6	4.241872	0.016605
Poly(A)-specific ribonuclease PARN	PARN	3.957987	0.005401
Endoglin	ENG	3.930967	0.043663
Ribosome biogenesis protein SLX9 homolog	SLX9	3.563605	0.008927
N-acetylglucosamine-6-sulfatase	GNS	3.560971	0.03549
Claspin	CLSPN	3.446785	0.018232
Active regulator of SIRT1	RPS19BP1	3.359693	0.02883
Pleckstrin homology-like domain family A member 1	PHLDA1	3.290734	0.008648
Bystin	BYSL	3.264988	0.01981
Ribosomal RNA processing protein 36 homolog	RRP36	3.230677	0.016246
Disks large-associated protein 4	DLGAP4	3.190853	0.007731
U3 small nucleolar RNA-associated protein 14 homolog A	UTP14A	3.188692	0.046915
Fibrillin-1	FBN1	3.162726	0.019768
Biogenesis of lysosome-related organelles complex 1 subunit 5	BLOC1S5	2.995847	0.032375
Glutathione S-transferase omega-1	GSTO1	2.970492	3.24E-06
39S ribosomal protein L51, mitochondrial	MRPL51	2.939908	0.036714
Follistatin-related protein 1	FSTL1	2.930418	0.010066
Thyroid transcription factor 1-associated protein 26	CCDC59	2.899679	0.033217
Uncharacterized protein C1orf131	C1orf131	2.880252	0.032046

28S ribosomal protein S6, mitochondrial	MRPS6	2.840361	0.006942
Ribosome maturation protein SBDS	SBDS	2.761412	0.016151
Metallothionein-2	MT2A	2.740393	0.01831
Transmembrane and ubiquitin-like domain-containing protein 2	TMUB2	2.724709	0.000824
Zinc finger CCHC domain-containing protein 9	ZCCHC9	2.710951	0.026735
Actin nucleation-promoting factor WASL	WASL	2.701133	0.007968
Serine protease 23	PRSS23	2.677409	0.011124
Ubiquitin-associated protein 2	UBAP2	2.657681	0.047239
H2.0-like homeobox protein	HLX	2.637583	0.008938
Twisted gastrulation protein homolog 1	TWSG1	2.573917	0.030983
Tissue-type plasminogen activator	PLAT	2.571593	0.015769
28S ribosomal protein S18c, mitochondrial	MRPS18C	2.563353	0.0102
Branched-chain-amino-acid aminotransferase, cytosolic	BCAT1	2.550797	0.006587
Lysosomal Pro-X carboxypeptidase	PRCP	2.527801	0.021718
cAMP-regulated phosphoprotein 19	ARPP19	2.475592	0.048011
Phosphoribosylformylglycinamidine synthase	PFAS	2.447184	0.038551
Tyrosine-protein kinase ABL1	ABL1	2.419443	0.025013
Purine nucleoside phosphorylase	PNP	2.39709	0.017725
Peptidyl-prolyl cis-trans isomerase FKBP1A	FKBP1A	2.367289	0.025215
Mth938 domain-containing protein	AAMDC	2.36717	0.00685
Metallothionein-1X	MT1X	2.365658	0.019215
Microtubule-associated protein 1A	MAP1A	2.364533	0.010687
RNA-binding protein 34	RBM34	2.354801	0.011065
Procathepsin L	CTSL	2.347498	0.03277
Copper transport protein ATOX1	ATOX1	2.344746	0.014571
Ankyrin repeat and KH domain-containing protein 1	ANKHD1	2.333067	0.047772
Glucosamine-6-phosphate isomerase 1	GNPDA1	2.329768	0.005076
Tissue factor pathway inhibitor 2	TFPI2	2.329683	0.032196
Cyclic AMP-dependent transcription factor ATF-1	ATF1	2.282305	0.046145
Proteasome subunit beta type-2	PSMB2	2.278764	0.010531
Sulfiredoxin-1	SRXN1	2.246201	0.037351
Cystatin-B	CSTB	2.1722	0.006847
MARCKS-related protein	MARCKSL1	2.150904	0.038411

Cyclin-dependent kinases regulatory subunit 1	CKS1B	2.120894	0.009877
Ribosome biogenesis protein WDR12	WDR12	2.115146	0.026121
D-aminoacyl-tRNA deacylase 1	DTD1	2.112355	0.046675
Glutaredoxin-1	GLRX	2.109265	0.01093
Transcription factor BTF3	BTF3	2.09818	0.039807
BH3-interacting domain death agonist	BID	2.089541	0.046592
Interferon-stimulated 20 kDa exonuclease-like 2	ISG20L2	2.06082	0.040107
Calmodulin-regulated spectrin-associated protein 2	CAMSAP2	2.046686	0.024982
Glutathione synthetase	GSS	2.041015	0.002476
Acyl-CoA-binding protein	DBI	2.008853	0.025212
Histone lysine demethylase PHF8	PHF8	0.486227	0.004505
Heat shock 70 kDa protein 1B	HSPA1B	0.480409	0.013242
Uncharacterized protein KIAA1671	KIAA1671	0.479972	0.011011
Collagen alpha-1(XII) chain	COL12A1	0.475553	0.021875
Coronin-1C	CORO1C	0.468651	0.024094
Pentraxin-related protein PTX3	PTX3	0.432968	0.003719
V-type proton ATPase subunit C 1	ATP6V1C1	0.425783	0.045193
Krueppel-like factor 4	KLF4	0.417	0.04096
Calcium/calmodulin-dependent protein kinase type II subunit gamma	CAMK2G	0.385685	0.034273
Histone-lysine N-methyltransferase EZH2	EZH2	0.349922	0.046092
Eukaryotic translation elongation factor 1 epsilon-1	EEF1E1	0.34687	0.04297
Cyclic AMP-dependent transcription factor ATF-3	ATF3	0.302078	0.03672
Endoplasmic reticulum chaperone BiP	HSPA5	0.301947	0.012761
Laminin subunit beta-2	LAMB2	0.30069	0.010928
Laminin subunit gamma-1	LAMC1	0.282319	0.002262
DnaJ homolog subfamily B member 9	DNAJB9	0.258984	0.000956
Guanine nucleotide-binding protein-like 3-like protein	GNL3L	0.251734	0.022801
RNA polymerase I-specific transcription initiation factor RRN3	RRN3	0.239496	0.041603
Telomerase Cajal body protein 1	WRAP53	0.233771	0.012588
Transcription initiation factor TFIID subunit 11	TAF11	0.22709	0.037376
Serine/threonine-protein kinase 31	STK31	0.134256	0.041105

Sulfhydryl oxidase 1	QSOX1	0.111336	0.002427
